# Emergence of ATP- and GTP-Binding Aptamers from Single RNA Sequences by Error-Prone Replication and Selection**

**DOI:** 10.1002/syst.202300006

**Published:** 2023-07-02

**Authors:** Falk Wachowius, Benjamin T. Porebski, Christopher M. Johnson, Philipp Holliger

**Affiliations:** a MRC Laboratory of Molecular Biology, Cambridge Biomedical Campus, Francis Crick Avenue, Cambridge CB2 0QH, UK

**Keywords:** RNA, aptamer, evolution, error-prone, quasispecies

## Abstract

The spontaneous emergence of function from diverse RNA sequence pools is widely considered an important transition in the origin of life. Here we show that diverse sequence pools are not a prerequisite for the emergence of function. Starting five independent selection experiments each from a single RNA seed sequence - comprising a central homopolymeric poly-A (or poly-U) segment flanked by different conserved primer binding sites - we observe transformation (continuous drift) of the seeds into low diversity sequence pools by mutation, truncation and recombination without ever reaching that of a random pool even after 24 rounds. Upon continuous error prone replication and selection for ATP binding we isolate specific ATP- or GTP-binding aptamers with low micromolar affinities. Our results have implications for early RNA evolution in the light of the high mutation rates associated with both non-enzymatic and enzymatic prebiotic RNA replication.

## Graphical abstract

**Figure:**
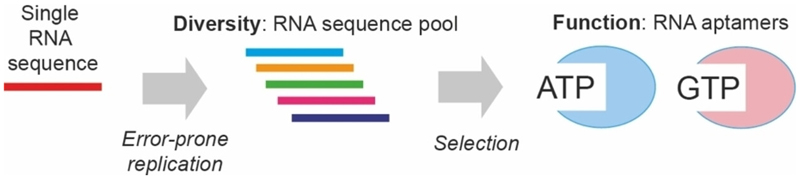

## Introduction

Compelling evidence supports the hypothesis of a primordial biology where RNA played a more central role. These include the central catalytic role of RNA in core genetic processes such as translation, RNA processing and splicing as shown by the ribozyme cores of the spliceosome, RNAse P and ribosomal peptidyl-transferase centre^[1]^ and the prevalence of nucleotide cofactors in core metabolism. Together with recent discoveries in prebiotic chemistry of varied and robust synthetic routes to the RNA (and DNA) nucleosides,^[2]^ their activation and their non-enzymatic polymerization outline an increasingly plausible path towards the emergence of an early RNA-based biology.^[3]^

A key transition along this path is thought to have been the spontaneous polymerization of activated RNA nucleotides into pools of random sequence RNA oligomers from which functional sequences could emerge. The generation of functional sequences from random pools has been recapitulated and studied by *in vitro* selection (SELEX) experiments,^[4]^ which have succeeded in the isolation of a diverse array of ligands (aptamers) and catalysts directly from diverse sequence pools of RNA, DNA and some unnatural nucleic acid analogues (XNA).^[5]^ Of particular interest in this context are nucleotide binding aptamers, which were among the first RNA aptamers to be isolated and which have been studied extensively as model systems. Such studies have sought to define the necessary and optimal size for binding as well as the sequence parameters necessary for aptamer discovery such as oligomer length, conformational pre-organization, pool complexity and adaptive landscapes.^[6]^ Extrapolation from such selection experiments and analysis of the mutational landscapes of functional oligomers suggests that the frequency of distinct ligand-binding motifs is low (< 10^−11^),^[4]^ even in highly diverse random sequence pools, although structural pre-organization can increase the chance of ligand discovery.^[6c]^ All of these experiments initiate from best-case scenarios regarding the size and sequence complexity of prebiotic oligomer pools, utilizing large, random sequence pools composed of approximately equal proportions of all four RNA nucleotides. However, studies on *ab initio* non-enzymatic polymerization of activated RNA nucleotides suggest that polymerization of longer RNA oligomers comprising all four RNA nucleotides in comparable proportions is disfavoured compared to the polymerization of homopolymeric or dinucleotide oligomers. This suggests that prebiotic oligomer pools might have been very significantly biased with regards to nucleotide composition^[7]^ with negative impacts on sequence and structural complexity and presumably phenotypic potential, i.e. the probability of functional sequences in a given sequence pool. Indeed, fundamental studies on ligase ribozymes suggest that function (at least in the case of catalysis) strongly scales with sequence complexity.^[8]^ These findings thus pose a challenge to the conjecture of spontaneous emergence. The probability of the latter would seem to depend critically on pool size and sequence complexity, as these parameters inform both the initial frequency of functional sequences and the ease (the number of evolutionary trajectories) by which functional sequences can be accessed from arbitrary points in sequence space.

Here we sought to critically test these arguments by exploring an extreme scenario of pool complexity: the emergence of functional RNAs starting from single, non-functional, predominantly homopolymeric RNA sequences under a high mutation regime mimicking the poor fidelity of prebiotic RNA replication. We performed five independent selection experiments starting each from a single, but distinct, partially homopolymeric RNA sequence (comprising a central 39nt poly-A or poly-U stretch flanked by different and conserved 15nt primer binding sites) and subjecting it to cycles of error prone replication and selection for ATP binding. We describe the discovery of both ATP- and GTP-binding RNA aptamers (from two independent selection experiments) and characterize their binding affinity and specificity.

## Results and Discussion

In a typical SELEX (Systematic Evolution of Ligands by EXponential Enrichment) experiment the sequence diversity is high at the beginning and is progressively reduced during each selection step. In contrast, here we start with a single “seed” sequence and introduce diversity before each selection round by error prone replication. Unlike the fully random sequence pool of a standard SELEX experiment, this creates a “cloud of genotypes” connected by mutation to a single “seed” sequence, akin to a quasispecies in viral replication. We sought to perform selections from sequences of minimal informational complexity, but purely homopolymeric sequences are challenging to propagate over multiple rounds of selection. We therefore settled for a compromise of partially homopolymeric seed sequences, whereby a central 39 nt homopolymeric stretch of polyA or polyU sequences is flanked on each side by different 15 nt conserved primer binding sites (comprising all four nucleotides in approximately equal parts). We chose poly-A and poly-U seed sequences to initiate selections as these may more accurately reflect a prebiotic scenario, as indicated by the generally increased yield and length distribution of poly-AU RNA polymers obtained by non-enzymatic non-templated polymerization compared to mixed sequences.^[7b,c]^ Furthermore, such sequences avoid the complications arising from the propensity of poly-G, poly-C sequences to form highly stable non-canonical secondary structures (such as G4 quadruplexes and i-motifs) that can hinder efficient replication.

**Figure 1:**
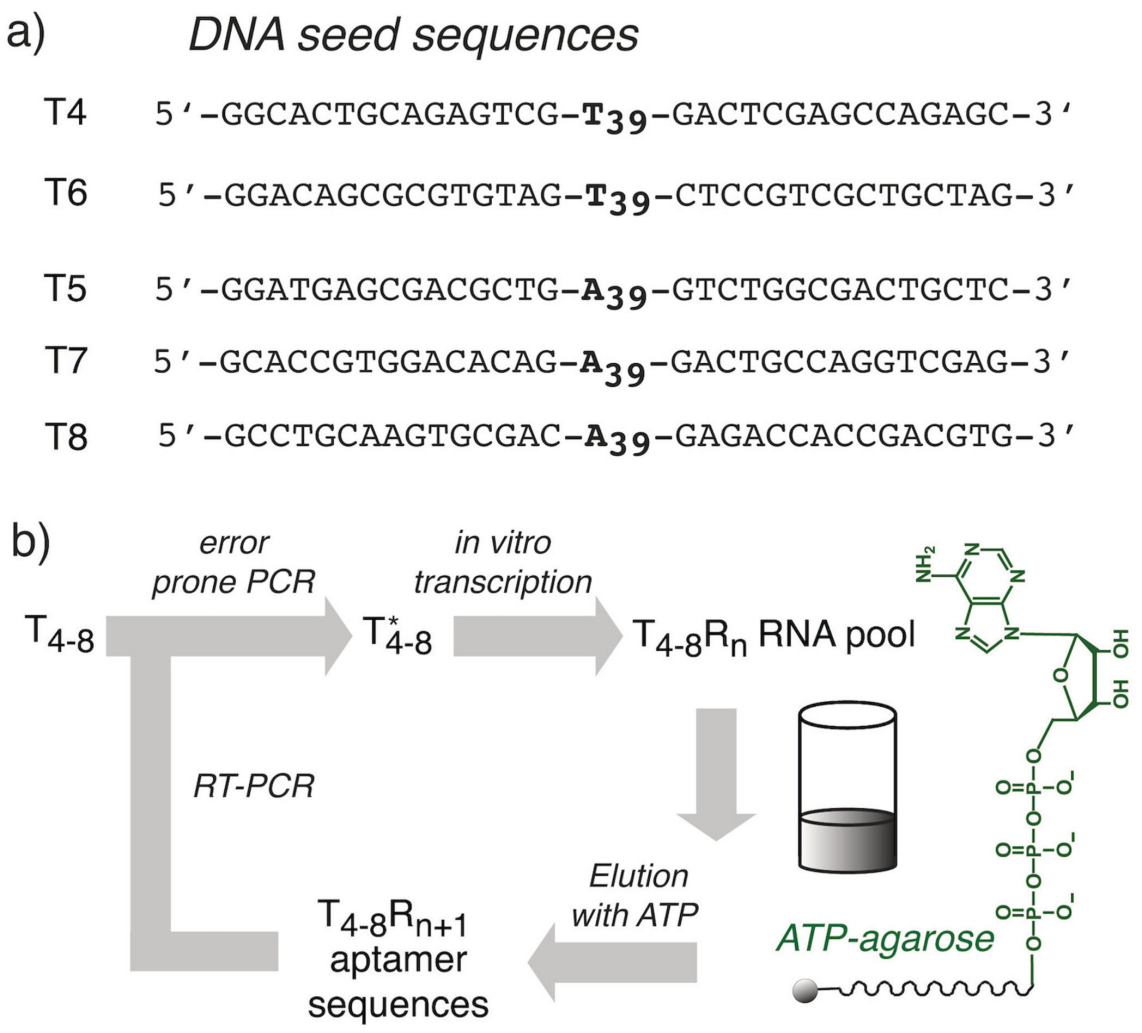
High mutation selection. Schematic representation of the high mutation selection scheme starting from single poly-T (T4, T6) or poly-A (T5, T7, T8) sequences (a) flanked by different primer binding sites. b) Principal workflow: T_4-8_ seed sequences are diversified by error prone PCR to yield sequence pool T*, which is transcribed into RNA, incubated with ATP agarose (γ-phosphate-linked), non-bound sequences (flow-through) are discarded and RNA sequences bound to the ATP matrix (ATP aptamers) are eluted with ATP. RNA sequences are reverse transcribed to cDNA and amplified by PCR (RT-PCR) and rediversified by error prone PCR for another round of selection.

Our experimental setup follows, with slight modifications, the canonical SELEX scheme for nucleotide aptamer selections^[9]^ (Figure 1). We carried out five independent selection experiments starting each from a single sequence (“seed”) with two sequences (T4, T6) with a poly-U segment and three sequences (T5, T7, T8) with a poly-A segment, each flanked by unique and different primer binding sites, to avoid cross contamination (Figure 1, Supplementary Table S1). Furthermore, unlike previous selection experiments that use C8-linked ATP agarose, we used ATP agarose with the ATP linked via the gamma phosphate.^[10]^

Our protocol includes an error-prone PCR (epPCR) step (according to standard protocols (0.2 mM NTPs, 1 mM MnCl_2_)^[11]^). We performed this epPCR step for every selection round including for the initial pre-selection repertoire (R0), to introduce sequence diversity. In contrast to the reported epPCR mutation rate (mean number of mutations per base pair (30 cycles)) of 0.66±0.13% (with a close to equal ratio (0.8) of transitions/transversions),^[11]^ we observe much higher mutation rates in particular for the generation of the 1^st^ round repertoire (R0). While an accurate mutation rate for R0 and for the other rounds is difficult to calculate due to pervasive slippage, truncation and recombination among amplified sequences (as is evident by the wide length distribution of R0 (and later round sequences) (Supplementary Figures S1–S4), due to a clear inherent bias of polymerases against complete replication of the homopolymeric stretch. Indeed, epPCR amplified sequence populations are rapidly dominated by very short (3–5 nt) polyA or polyT sequence inserts and even after the introduction of size selection by gel purification, we find that the first rounds of selections are dominated by short homopolymeric sequences (Figures S1–S4). While the “tyranny of the short motif”^[12]^ is known to favour “parasitic” (i.e. short, non-functional and fast replicating) sequences selection experiments, the homopolymeric sequence context adds additional bias and opportunities for template switching and recombination in PCR.^[13]^

Given these complications, we sought to estimate error rates by calculating the Damerau-Levenshtein distance (LD) of sequence populations R0, R1 etc. to the original “seed” sequence. LD between two nucleic acid strings is defined as the minimum number of operations (comprising mutations, insertions or deletions of a single base, or transposition of two adjacent bases) required to change one string into the other. Given the high levels of slippage and truncation observed in epPCR of homopolymeric seed sequences, LD provides a more accurate measure of mutational distance than the more commonly used Hamming distance in the sequence pools. We find LDs for the starting pools T5R0 of ~7 (for T5R0) and ~4 for T7R0 and T8R0 suggesting estimated average mutation rates of 10%–18% per base pair (including substitutions, indels as well as transposition mutations) in the homopolymeric stretch (Supplementary Figures S5,S6). Thus on homopolymeric sequence templates epPCR error rates are increased by > 10-fold creating a quasispecies sequence pool at an average mutational distance of ~4–7 from the seed sequence. However, this estimate needs to qualified by the fact we observe an aggregate error rate of reverse transcription and epPCR (and Next generation (Illumina) sequencing). Should the reverse transcription and Illumina sequencing steps be equally more error prone on polyA/polyU stretches, the measured LD of the RNA populations in R0 (and later), may be an overestimate. Thus, the diversity of the R0 starting pools is very significantly lower than the commonly used random sequence pools of standard SELEX experiments both judged by LD (LD for a random pool ~ 29–30) as well as by visual inspection of the most common sequences, which in early rounds are predominantly truncated polyA stretches (Supplementary Figures S1–S4). Indeed, in no experiment do the pools generated by iterative mutation and selection reach the sequence diversity of a random pool (even after 24 rounds (Supplementary Figures S5,S6)

To investigate if functional RNA sequences could be isolated from these pools we performed a further 8 rounds of selection for binding to ATP-sepharose and error prone PCR amplification (Figure 1) for T5, 16 rounds for T7 and T8 (with increased selection stringency after round 12 (Materials & Methods)) and 24 rounds for T4 and T6.

**Figure 2:**
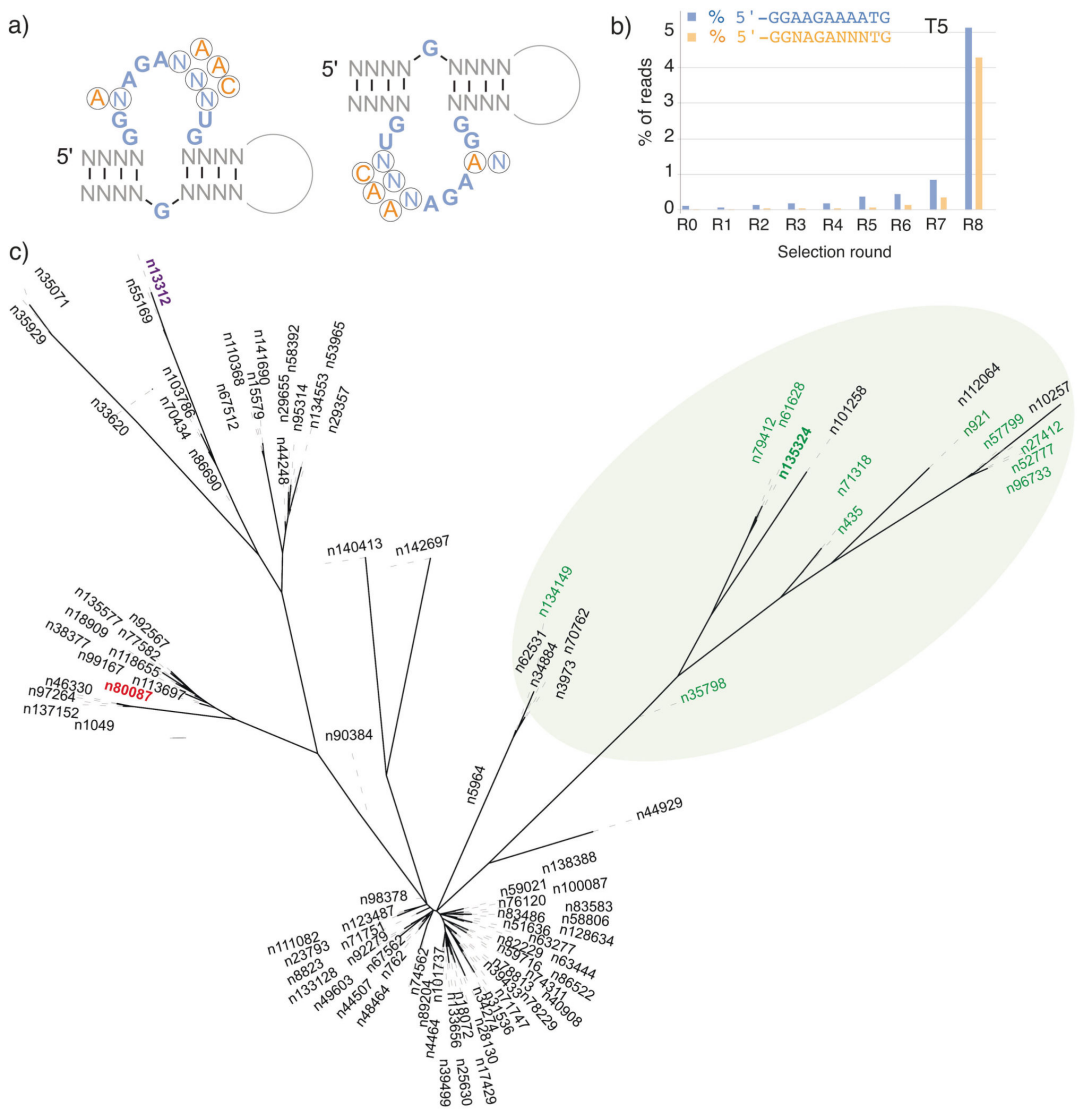
Emergence of ATP aptamer motifs. a) Canonical (left) and reverse ATP aptamer motif (right) with conserved bases in blue (canonical motif) and orange (reverse motif). N-NNN non-essential loop residues are circled. b) Ratio (%) from total unique sequences of the minimal (GGNAGANNNTG (N-NNN) and near canonical GGAAGAAAAUG (A-AAA) ATP aptamer loop motif for rounds R0-R8 of the T5 selection. c) Phylogenetic tree of the ATP aptamer motifs in T5R8 sequence pool (perfect match motifs in green). Note the canonical ATP aptamer loop/G-bulge motif appears in all, with variations confined to the stem regions (see Supplementary Figures S8, S9).

Next, we searched all selections for appearance of the canonical RNA ATP aptamer loop motif,^[9]^ which had previously been observed multiple times in unrelated selections.^[14]^ Said motif comprises a conserved stem-loop-structure with an essential bulged G opposite the recognition loop (Figure 2a). Only 7 of the 11 nucleotides in the purine-rich recognition loop are conserved (5’-GGNAGANNNTG), while even individual mutations of this conserved residues do not completely abolish binding. While the stem regions are essential, their composition has only minor influence on aptamer function, however at least one GC base pair in each stem is likely preferred for the formation of stable stems. We observed only sporadic appearance of the motif in the poly-U (T4, T6) selections even after 24 rounds of selection. In contrast, in poly-A (T5, T7, T8) selections we observed an increase of both the relaxed (5’-GGNAGANNNTG-3’) (N-NNN) and the perfect ATP recognition loop ((5’-GGAAGAAAATG-3’ (A-AAA)) in T5R8 (Figure 2b) and the T7-, T8R12 pools (Supplementary Figure S7), after which frequency dropped, possibly related to the more stringent selection conditions for the final rounds of T7 and T8 selections. Encouraged by this, we next searched for perfect (or near perfect) matches for the canonical ATP binding motifs (5’-GGAAGAAACTG-N_n_-*G*-3’; 5’-*G*-N_n_-GGAAGAAACTG-3’; including the bulged *G*) by leveraging the search algorithm of Luptak and colleagues.^[15]^ While no unambiguous motif hits were found in the T7R12 and T8R12 selections (despite appearance of the recognition loop sequence), in the T5R8 selection, we obtained > 100 hits with divergent stem sequences (Figure 2c, Supplementary Figure S8) (of which 15 comprised a perfect match with the canonical motif). Several of these showed predicted canonical (or near canonical) ATP aptamer secondary structure motifs (as judged by RNAfold^[16]^) (Supplementary Figure S9).

**Figure 3:**
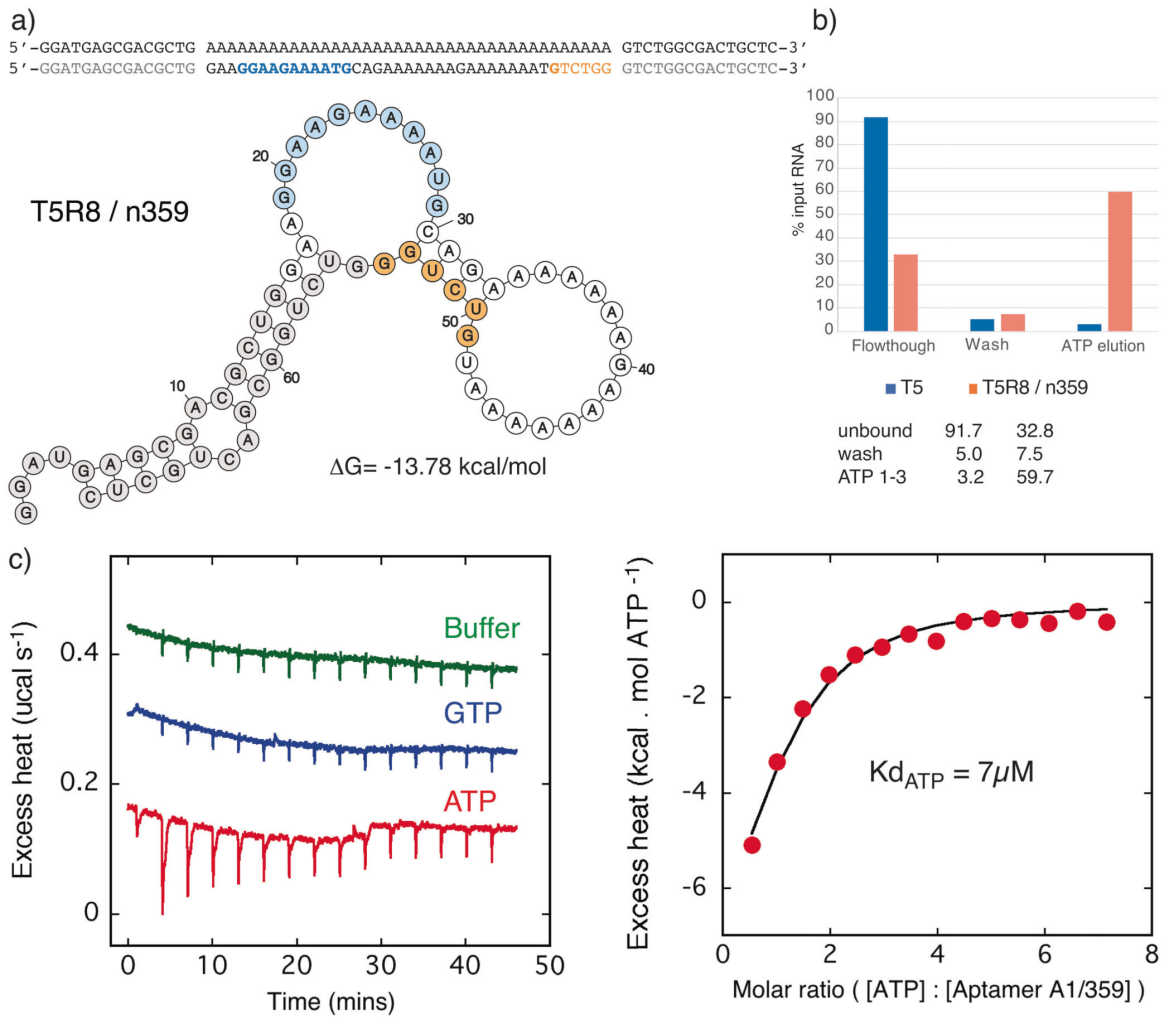
ATP Aptamer T5R8/359. a) Sequence and predicted secondary structure. T5R8/359 aptamer vs T5 seed sequence with conserved primer sequences (grey), nucleotides corresponding to the canonical ATP aptamer loop motif (bold) with loop (cyan) and nucleotides deriving from partial duplication of the primer sequence (orange) are shown. b) ATP-agarose column elution of T5 seed sequence (blue) and the selected ATP aptamer T5R8/359 (orange). c) ITC binding experiments : raw data showing additional binding heat for ATP titration compared to GTP or buffer controls (left panel) and integrated excess heat of ATP titration fit to a simple one site binding model (right panel).

**Figure 4:**
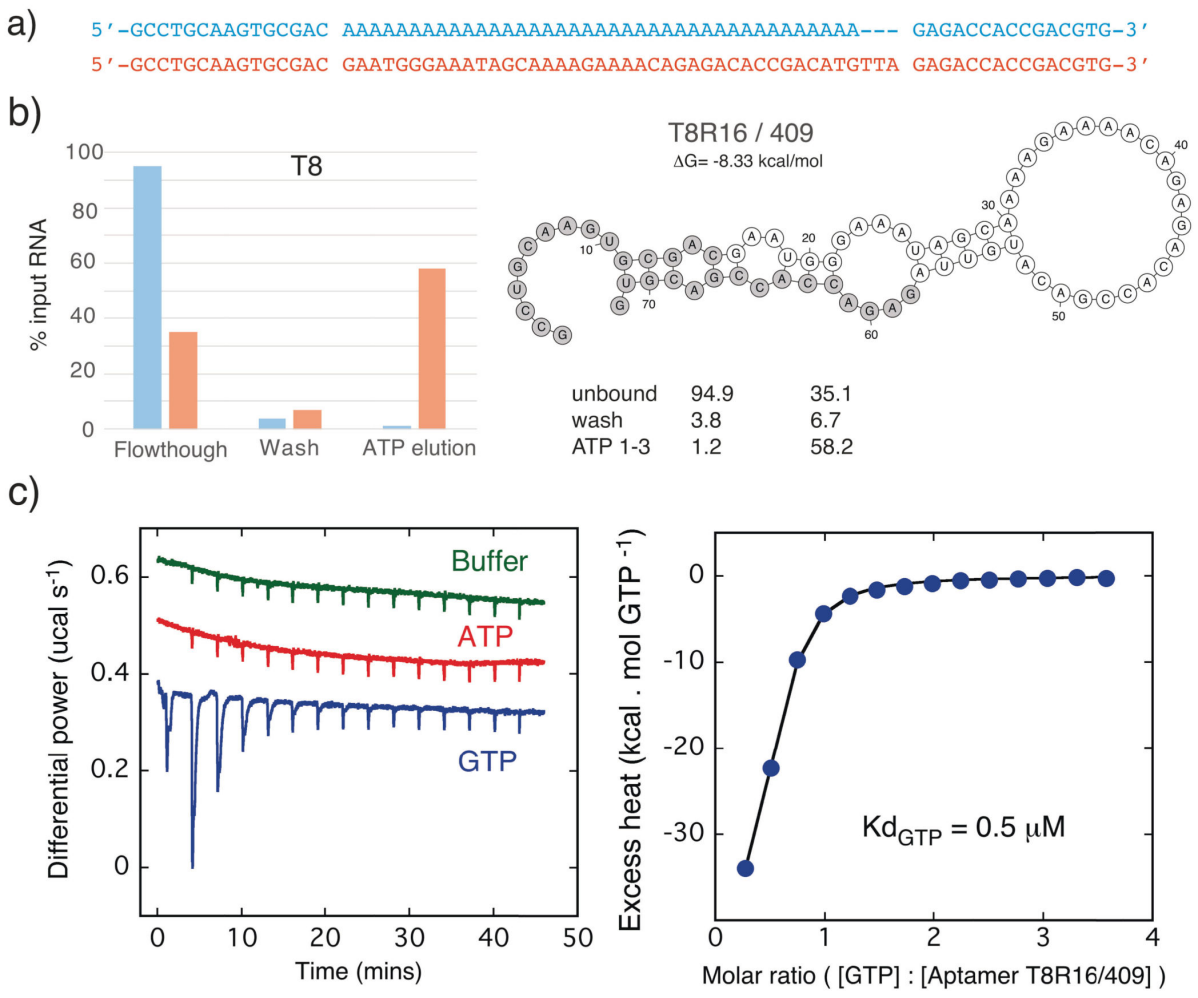
GTP Aptamer. a) T8 seed sequence (blue) and selected aptamer T8R16/409 (orange), b) respective ATP-agarose column elution T8 seed vs T5R8/359 (left) and predicted T8R16/409 secondary structure (RNA fold^[16]^) with primer sequences (grey) (right). Deletion of primer sequences yields truncated aptamer T8R16/409core, which retains NTP binding activity as judged by column binding and elution (see Supplementary Figure S11). c) ITC raw data for T8R16/409 showing additional binding heat for GTP titration vs. ATP or buffer controls (left) and integrated excess heat of GTP titration fit to a simple one site binding model (right).

As perfect matches to the canonical ATP aptamer motif had previously been shown to invariably bind ATP,^[15,17]^ we chose examples of more divergent, but highly enriched sequences from both the T5 and the less promising T4, T6, T7 and T8 selections and compared their ATP binding activity to their respective seed sequences by ATP-agarose column elution. Among these we identified several motifs with putative ATP binding activity as judged by column elution profiles (T4R24/377, T5R8/359, T6R20/388, T7R16/401, T8R16/409) (Figure 3a, Figure 4b, Supplementary Figure S10, Supplementary Table S2).

However, among these only T5R8/359 and T8R16/409 showed strong ATP binding compared to their seed sequences and we focused further analysis on these two motifs. T5R8/359 comprises an extended 12 nt purine rich ATP recognition loop flanked by two dsRNA regions and an GG bulge opposite the loop (Figure 3b), thus differing by just 2 mutations from the perfect, canonical ATP aptamer motif. The essential opposing bulged G appears to originate from a partial (6 nt) duplication of the 3’ primer sequence (Figure 3b) likely originating from a PCR template switching/recombination event. In concordance with this T5R8/359 without the 6 nt 3’-primer insert (T5R8/359Δ6) showed no ATP binding as judged by column elution. (Supplementary Table S2). Despite an unpaired G:A next to the canonical G-bulge, T5R8/359 appears to be able to adopt a canonical ATP aptamer-like fold (as judged by ATP binding), providing a variation on the ATP binding motif. Where previously, a single G-bulge was considered essential for ATP binding,^[9,17a]^ T5R8/359 suggests that an expanded loop and double G–G-bulge also support formation of a specific ATP binding pocket. This relaxes the search criteria for ATP aptamer motifs and suggest that there may be even more ATP-binding aptamers in the T5R8 pool than the > 100 canonical motifs detected (Figure 2c, Supplementary Figure S8) and that more relaxed search criteria might be applied to identify ATP aptamer motifs in both SELEX experiments and in genomic RNA pools.^[15,17b]^

T8R16/409 from the T8 selection also showed clear ATP binding as judged by column elution (Figure 4a, b) despite showing no sequence (or secondary structure) similarity with the canonical ATP aptamer motif. Notably, truncation by deletion of either (620) or both (T8R16/409core (105)) primer sequences resulted in no difference in ATP-binding as judged by column elution profiles, while the deletion of a central stretch of nucleotides abolished binding. While CTP and UTP elution profiles were similar to ATP, 409core appeared to be more readily eluted with GTP (Supplementary Figure S11, Supplementary Table S3), pointing towards specificity for GTP.

Next, we sought to measure specific binding affinities of these two most promising aptamer sequences T5R8/359 and T8R16/409 by isothermal titration calorimetry (ITC). Binding experiments were performed three times with similar results. The fitted isotherm indicated a Kd of aptamer T5R8/359 binding ATP of around 7 μM (Figure 3c). In contrast, aptamer T8R16/409 showed GTP binding with a more than 10x higher affinity with a Kd around 0.5 μM (Figure 4c). Both aptamers showed remarkable selectivity with no GTP binding detectable for T5R8/359 and no ATP binding detectable for T8R16/409. Indeed the observed integrated heats were identical to those obtained by titrating the nucleotides into buffer alone suggesting no interaction at a level detectable in these experiments. This is especially striking for T8R16/409, which showed clear and specific GTP binding despite selection on an ATP-agarose resin and clear binding to and elution from the resin by ATP (as well as other ribonucleotides)) (Supplementary Figure 10). As column elution assays can detect binding affinities in the mid to high μM range, this may simply reflect a promiscuous, but lower affinity of T8R16/409 for ATP (and indeed other nucleotides)(Supplementary Figure S11). The GTP binding curve of T8R16/409 yielded a reliable fit to a stoichiometry of around 0.5. This underestimate from an expected 1:1 value probably reflects potential errors in the concentrations of both active aptamer and nucleotide, which were not easy to quantify, as well as the possibility of unstructured or misfolded aptamer material in the experiment. In light of the observed value for GTP we constrained the stoichiometry at this same value of 0.5 when fitting the ATP data. The issue of exact active concentration of materials used introduces some additional uncertainty into the precise values of the dissociation constants (Kd) measured. However, the experiments clearly demonstrate the binding of nucleotides by both these aptamers and their ability to discriminate between ATP and GTP in the μM and sub-μM regime of affinities.

## Discussion

Here we have described the discovery of specific ATP- and a GTP-binding RNA aptamers starting in each case from a single, non-functional seed sequence subjected to a high mutation regime and selection. In two of five selection experiments we discover nucleotide binding aptamers with low μM binding affinities for either ATP or GTP (as measured by ITC) (Figures 3, 4) as well as potentially hundreds of *bona fide* ATP aptamers as judged by the appearance of the canonical ATP aptamer motif (Figure 2, Supplementary Figure S8). Thus, the emergence of functional RNAs is not dependent on highly diverse, random sequence pools as a starting point. Function can arise even from single sequences with low informational and molecular complexity (> 50% poly-A or poly-U) under a high mutation regime.

**Figure 5:**
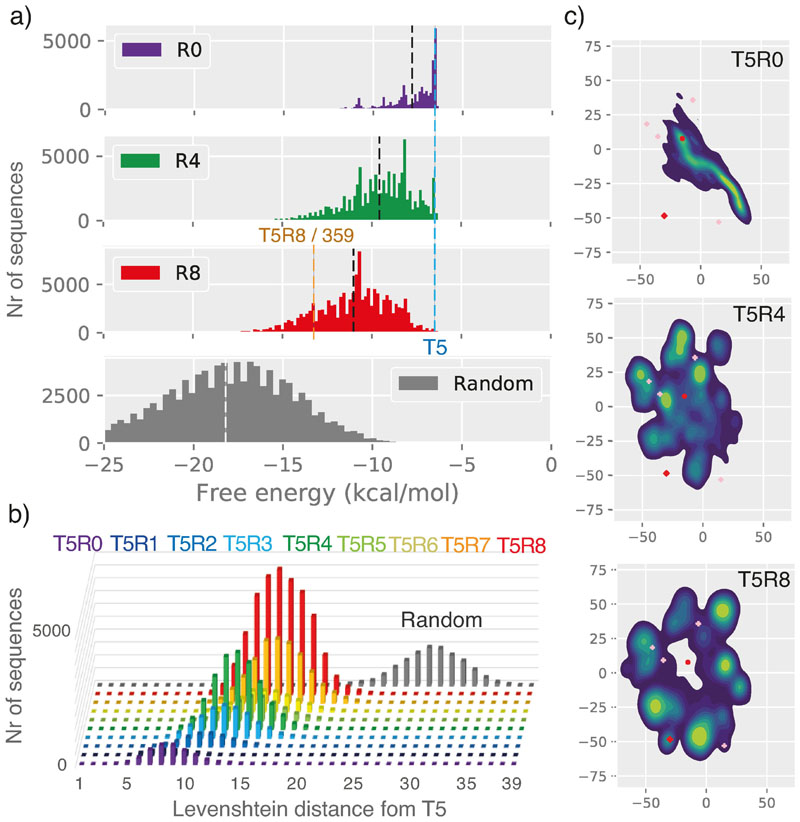
Sequence pool development during selection. a) (left panel) RNA folding energy distributions for T5R0 (purple), R4 (green), R8 (red) compared to random sequence pools (median: black/white dotted line). (right panel) b) Levenshtein distance distribution of T5 sequence pool over the course of selection (R0-R8) with reference to a random sequence pool (grey). c) Sequence space evolution as shown by t-stochastic neighbour embedding (tSNE) for T5R0, R4 and R8 (T5 seed sequence (red dot), T5R8/359 aptamer (red diamond), ATP aptamer motif perfect hits (Figure 2, pink crosses)(see Supplementary Figure 13 for full trajectory).

The emergence of new function by iterative cycles of mutation and selection from a single (or very few) starting sequences has precedents in biology e.g. in virology (quasispecies),^[18]^ the avian immune system and in hypermutating B-cell lines.^[19]^ Laboratory examples also include the evolution of function (phage infectivity) from an arbitrary soluble polypeptide sequence^[20]^ as well as model experiments in the ribozyme field morphing one ribozyme function into another.^[21]^ However, these examples differ from our work in that new functions emerge from already soluble (or stably folded) and functional protein (or RNA) domains. In contrast, we consciously chose non-functional starting sequences with low informational complexity (Figure 1) comprising a central homopolymeric stretch of A_39_ or T_39_ (U_39_), which induced high initial mutation rates due pervasive polymerase slippage on the homopolymeric sequences. Initial diversified R0 pools generated this way comprise a large number of truncated sequences radiating out from the seed by an average Levenshtein (LD) distance of 4–7 (T5, T7, T8) (Supplementary Figures S1-S6). As selection rounds progress, mutation rates per round are reduced as sequence pools diversify away from homopolymeric stretches. However, even though the sequence and hence molecular diversity of the pools in all selections continued to expand during the selection process, they never reached the complexity of a fully random pool and thus maintain a significant sequence bias and imprint of the seed sequence even after 24 rounds of mutation/selection (Supplementary Figures S5, S6). Nevertheless, new sequence features continued to emerge during the course of selection as shown by principal component analysis by t-stochastic neighbour embedding (tSNE)^[22]^ (a two-dimensional projection visualization of sequence space evolution) (Figure 5, Supplementary Figures S12, S13).

While high mutation rates lead to rapid diversification, it has been argued that they may hinder the fixation and stable inheritance of a given phenotype. Such high mutation regimes might bias pool adaption towards small motifs,^[23]^ and/or towards RNA folds that are both abundant and robust (stable) (such as hairpin and hairpin-like motifs).^[24]^ Such structurally stable motifs can better withstand disruption through high mutation rates even if they do not present the most “fit” species, i.e. best adaptive solutions in the pool, in a process which has been termed “survival of the flattest”.^[25]^ Indeed, in all cases overall selected sequence pools become more structured and more stably folded than starting seed sequences (as judged by secondary structure prediction) (Figure 5, Supplementary Figures S11). However, in no case do they reach the median stability of a random pool. The most parsimonious explanation is therefore that this trend may simply reflect increasing sequence diversification, although we cannot rule out some degree of stability selection. If present, its contribution is likely to be small, as all selections yield stably folded motifs with detectable, if weak, ATP binding, as judged by column elution (Supplementary Figure S4, Supplementary Table S2). The folding stabilities of both weakly ATP binding (as judged by column elution (Supplementary Figure S10, S11, Supplementary Table S2)) as well as highly functional aptamer binding motifs (T5R8/359, T8R16/409) show no clear bias towards stability significantly higher than the median pool stability for their rounds of emergence (Figure 5a, Supplementary Figure S11). While structural pre-organization has been shown to enhance the success of aptamer discovery,^[6c]^ and may buffer a phenotype against high mutation rates, here the extent of folding and structural stabilization does not appear to correlate strongly with function.

The emergence of the canonical ATP aptamer recognition loop motif in several separate selections (from distinct seed sequences) suggests that it may constitute privileged molecular solution for ATP binding. Indeed, it has been previously discovered multiple times in independent selection experiments including for binding to ATP containing cofactors such as NAD^[14a]^ and SAM^[14b,26]^ and was furthermore identified in several bacterial and eukaryotic genomes.^[15,17b]^ This parallels the case of the Hammerhead ribozyme motif^[27]^ and suggests that both of these motifs may represent minimal optimal solutions in RNA sequence space. The isolation of the canonical ATP aptamer motif in the T5 selection may also indicate its resilience to high mutation rates. The isolation of a GTP binding aptamer (T8R16/409) (Figure 4) from an ATP selection experiments may seem fortuitous, but is less surprising considering the previous discovery of numerous GTP binding aptamers at a relatively short mutational distance from the canonical ATP aptamer motif.^[28]^ Furthermore, GTP aptamers appear to be more structurally and functionally diverse and may therefore also be more abundant in sequence space. Indeed, “CA” repeats as short as 3 nucleotides^[29]^ or G-quadruplex motifs^[30]^ have been reported to bind GTP. Finally, while the GTP binding aptamer only binds GTP in solution (as judged by ITC), it clearly binds to the ATP-agarose resin (both as the full-length T8R16/409 aptamer as well as a truncated core motif (T8R16/409core) (Supplementary Figure S11, Supplementary Table S3).

Our results also underline the importance of sequence context and scaffolding in adaptive outcomes, as the same 39 nt poly-A seed sequence evolved very differently depending on the conserved 15 nt flanking sequences (which are not mutated during selections). While the purine-rich ATP aptamer loop motif was enriched in all poly-A selections (T5, T7, T8) (Figure 2, Supplementary Figure S7), the key opposing G-bulge required for high affinity ATP binding motif only arose in the T5 selection deriving from a partial duplication of the 3’ flanking sequence. Furthermore, the variable success of aptamer discovery may reflect the availability of accessible vs. inaccessible adaptive trajectories e.g. through formation of favourable RNA secondary structures.^[31]^ Indeed, (although not intentionally designed by us) the starting sequence of the successful T5 selection shows a higher degree of pre-organization (as judged by folding energy (ΔG=−7.3 kcal)) than the comparable T7 and T8 poly-A seeds (Figure 5a). However, it is notable that while the T8 selection did not yield the canonical ATP aptamer fold, it was successful in yielding an ever higher affinity GTP-binding aptamer despite the lowest degree of initial secondary structure or folding stability of any seed sequence (ΔG=−2.3 kcal), while at the same time the unsuccessful T4 and T6 poly-U seeds showed the highest and third highest structural preorganization ((ΔG=−9.6/−7.2 kcal) (Supplementary Figure S11). Thus, while structural preorganization undoubtedly can aid aptamer discovery,^[6c]^ it may not by itself be sufficient for success.

On the other hand, the failure of the T4 and T6 poly-U seeds to yield high affinity ATP binding aptamers (even after 24 rounds of selection) may simply be a reflection of the radically different mutational distances of the seed sequences from adaptive peaks. Indeed, the canonical purine-rich ATP binding motif^[9]^ is more readily accessible from poly-A than poly-U, not only with regards to mutational distance but also due to the increased likelihood of A > G transition vs. U > A/G transversion mutations in mutagenic PCR. Interestingly, this dichotomy is a reflection of the SELEX process comprising transition between RNA and DNA intermediates (through reverse transcription into cDNA, PCR amplification followed by in vitro transcription), whereby only one RNA strand (+) is available for selection. In a prebiotic scenario, where RNA would be replicated from RNA templates (either non-enzymatically or by RNA polymerase ribozymes),^[3]^ both RNA (+) and (−) strands would potentially be available for selection and poly-A- and poly-U-rich seed sequences would therefore provide comparable starting points for evolution.

## Conclusions

In biology, with the exception of specialized tissues like the immune system^[19]^ and some viruses,^[18]^ genetic information is replicated with high fidelity and the main sources of genetic diversity are drift (a slow accumulation of nearly neutral mutations) and recombination. In contrast, current model systems of prebiotic RNA replication suggest a highly error-prone process both for non-enzymatic RNA replication^[32]^ as well as enzymatic replication by polymerase ribozymes^[33]^ combined with an innate tendency of RNA oligomers for recombination.^[34]^ The high mutation regime approach described herein seeks to mimic such a scenario. Encouragingly, our results indicate that highly error-prone replication does not impede the emergence of simple functional RNA motifs and may aid or even be required for their emergence from the likely compositionally biased prebiotic RNA pools^[35]^ of limited sequence diversity or indeed from single RNA sequences.

## Experimental Section

### Oligonucleotides

DNA and RNA oligonucleotides (listed in Supplementary Table S1) were from Integrated DNA Technologies and if necessary were purified, by denaturing PAGE (8 M urea, TBE). The 2 poly-T (,T4, T6) and 3 poly-A (T5, T7, T8) starting sequences consist of 69 nt, containing an either 39 nt poly-A or poly-T sequence flanked by different 15 nucleotide (nt) primer binding sites on both ends (Supplementary Table S1). The sequences were designed with nonoverlapping primer binding sites to avoid cross contamination from different evolution-selection experiments.

### Evolution-selection experiments

The poly-A/poly-T DNA sequences (T4, T5, T6, T7, T8) were PCR amplified (MJ Research Tetrad PTC-225 Thermal Cycler) (25 cycles), using the OneTaq hot start master mix (Promega) and corresponding primers (Supplementary Table S1), with the forward primer including the T7 RNA polymerase promoter sequence. PCR products were gel purified by agarose gel electrophoresis and isolated applying QIAquick columns (Qiagen), followed by in-vitro transcription applying the Megashortscript kit (Ambion). After DNase I (Ambion) treatment (30 min, 37°C), the RNA was isolated from the reaction mixture using the RNeasy kit (Qiagen) and the obtained crude RNA was purified by denaturing PAGE (8 M Urea, TBE).

The RNA sequences were first subjected to a negative selection step on sepharose, before selecting against ATP agarose. For the selection step 10 pmol of purified RNA was heated in reaction buffer (300 mM NaCl, 5 mM MgCl_2_, 20 mM TRIS pH 7.4) to 65 °C for 5 min and cooled down at rt for 20 min. The RNA was added to 80 μl of sepharose (Sigma), that was prewashed with reaction buffer (3×160 μl), in Costar microfilter spin columns (0.45 μm, CA membrane, Corning), and was incubated for 1 h at room temperature (rt). The flow-through was subsequently added to 40 μl of reaction buffer prewashed (3×120 μl) ATP agarose (γ-phosphate-linked, 8−12 μmol/ml, Innova Biosciences) in Costar microfilter spin columns and incubated for 1 h at room temperature. The flow-through was discarded and the ATP agarose resin was washed three times with 160 μl of reaction buffer, followed by the addition of 120 μl of 5 mM ATP (Roche) in reaction buffer and incubated for 30 min.

For the selection rounds 12–16 (T7, T8) and 20–24 (T4, T6) the columns were first eluted with 1 mM ATP in reaction buffer, followed by the 5 mM ATP elution. The flow-through was desalted using Vivaspin 500 (3000 MWCO) concentrators (Sartorius). The obtained RNA was reversed-transcribed and PCR-amplified using the Superscript III One Step RT-PCR system (Invitrogen) followed by an additional error-prone PCR step (20 cycles) using standard Taq DNA polymerase (HT Biotechnology) with equimolar concentrations of dNTPs (200 μM) but with the supplement of 1 mM MnCl_2_ (final conc.). The obtained DNA was purified and in-vitro transcribed to RNA as described above and the purified RNA was added into the next selection cycle. The sequences defined as round 0 corresponds to one round of standard PCR (25 cycles) and one round of error-prone PCR (20 cycles).

### Deep Sequencing

DNA products from different selection rounds were PCR amplified using extended primers including adaptor sequences for Illumina sequencing (Supplementary Table S1). The DNA was purified by agarose gel electrophoresis and extracted using the Qiagen gel extraction kit (Qiagen), followed by quantification by q-PCR (Brilliant III Ultra-Fast SYBR® QPCR, Agilent). Sequencing was performed on a MiSeq Illumina platform and sequencing data were processed by trimming of adaptor sequences, quality filtering, conversion to FASTA format and collapsing of sequences according to abundance using the Galaxy platform web server: https://usegalaxy.org. The occurrence of the ATP aptamer loop motif 5’-GGAAGAAAATG-3’ or 5’-GGNAGANNNTG-3’ was analysed using FIMO (Find Individual Motif Occurrences) http://meme-suite.org/tools/fimo. RNA secondary structure prediction and display was carried using the ViennaRNA package 2.0,^[16]^ specifically RNAfold either with or without aptamer loop motif constraints.

#### Mutation rate analysis

We measured the mutation rate during error prone PCR (epPCR) through deep sequencing of round 0 (R0), which is the unselected output from epPCR of a poly-A stretch of 39 nucleotides. The mutation rate for each position was calculated with equation 1.

(1) $$M_{i}=1-\frac{C_{i}}{T_{i}}$$

Where C_i_ is the number of correct nucleotides (A) at position i, and T_i_ is the total number of nucleotides at position i. The average mutation rate is the mean rate of all 39 nucleotides.

#### Levenshtein distance analysis

To compare converging or diverging sequence populations against a target sequence, we used the *Levenshtein* distance, which was calculated by counting the number of substitutions, deletions and insertions required to convert a given sequence to the reference sequence using the Wagner-Fischer algorithm.^[36]^

#### Sequence space projections

To visualise sequence space of our aptamer selection experiments, we chose to reduce the dimensionality and project it into two dimensions using t-Stochastic Neighbour Embedding (t-SNE)^[22]^ implemented in the openTSNE package v.0.4.0.^[37]^ Here, we used a perplexity of 50, a cosine metric, and principal component analysis for initialisation, with all other parameters as default. We used sequencing reads that were trimmed to contain the full 5’- and 3’-primer sequences, one-hot encoded into a binary triplet encoding, and padded with zeros out to 50 nucleotides. For the projection figures, we merged and collapsed reads across all rounds, fit the t-SNE model as described above, and transformed the reads for each round to draw a mixed scatter and kernel density plot using matplotlib^[38]^ and seaborn (https://doi.org/10.5281/zenodo.592845).

# Canonical ATP aptamer motif strict

h1 s1 h2 s2 h2’ s3 h1’

h1 0:0 NNNN:NNNN

h2 0:0 NNNN:NNNN

s1 0 GGAAGAAACTG

s2 0 N[30]N

s3 0 G

# Canonical ATP aptamer motif relaxed

h1 s1 h2 s2 h2’ s3 h1’

h1 0:0 NNNN:NNNN

h2 0:0 NNNN:NNNN

s1 0 GGAAGAAANTG

s2 0 N[30]N

s3 0 G

#### Phylogenetic tree generation

Canonical ATP aptamer motifs were identified using RNABOB as described above and a multiple sequence alignment performed with muscle v3.8.1551 using default settings.^[39]^ With the aligned motif sequences (including 5’- and 3’-primer sites), a phylogenetic tree was calculated with MrBayes 3.2.7 single default parameters.^[40]^ Phylogenetic trees (Figure 2, Supplementary Figure S8) were displayed using the EMBL iTOL online tool for the display, annotation and management of phylogenetic trees: (https://itol.embl.de/).

### Column and elution binding assay

RNA Sequences for the column binding assay, were generated by in vitro transcription from DNA templates generated by PCR amplification or by fill-in with primer TxT7 (Supplementary Table S1) using GoTaq (Promega). DNA was purified using QiaQuick (Qiagen), transcribed using the Megashortscript kit (Ambion) and purified using RNeasy (Qiagen). Cy5 labelled RNA was ordered purified (HPLC) from IDT. 40 μl ATP agarose was added to spin columns (0.45 μm, CA membrane, Corning) and equilibrated through washing with 3×120 μl buffer (300 mM NaCl, 5 mM MgCl_2_, 20 mM TRIS pH 7.4). 4 pmol RNA oligonucleotide in 80 μl buffer was heated to 65 °C, cooled down for 15 min, added to the column and incubated for 1 h at rt. After washing with 80 μl buffer, three to five subsequent ATP elutions (80 μl of 5 mM ATP in buffer) were performed, with a delay time of 5 min between each elution and the volume of the different fractions was reduced to ~15 μl by speed-vac. After the addition of 30 μl of 8 M urea/0.1 M EDTA the fractions were added to a 8 M urea PAGE gel and were run at constant power (30 W). The gel was stained by SYBR gold, analysed by a Typhoon scanner and quantified using ImageQuant (GE Healthcare). Binding assays were repeated 2–3 times for each RNA sequence.

### Isothermal titration calorimetry

ITC experiments were performed at 25 °C in 50 mM HEPES, 100 mM NaCl, 5 mM MgCl_2_, pH 7.6 buffer using a Malvern Panalytical both manual and auto iTC200 instruments. The RNA aptamers were loaded into the ITC cell at 6 μM and the nucleotides titrated from the syringe at 100–200 μM depending on affinity. Titrations were performed with 14 injections of 2.8 uL of nucleotide preceded by a small 0.5 uL pre injection that was not used during analysis. After baseline fitting and integration of excess heats the data were corrected using control heats observed for identical titrations of nucleotide solution into buffer. These control heats were small and similar to values obtained for buffer blank titrations. Corrected titrations were fit using Malvern Panalytical PEAQ software using a simple one set of sites binding model.

## Supplementary Material

Supporting Information

## Data Availability

Custom scripts used for analysis in this study can be found at GitHub:: https://github.com/holliger-lab/randomer-analysis

## Data Availability Statement

The data that support the findings of this study are available from the corresponding author upon reasonable request.
