# Supplementary material for: Emergence of ATP- and GTP-Binding Aptamers from Single RNA Sequences by Error-Prone Replication and Selection
Source: ChemSystemsChem. Author manuscript; Available in PMC 2023 Dec 9. (PMC7615363; doi:10.1002/syst.202300006)
Supplement: Supporting Information [file EMS191864-supplement-Supporting_Information.pdf]

ChemSystemsChem

Supporting Information

# Emergence of ATP- and GTP-Binding Aptamers from Single RNA Sequences by Error-Prone Replication and Selection

Falk Wachowius, Benjamin T. Porebski, Christopher M. Johnson, and Philipp Holliger*

**Supplementary Information:**

# Emergence of ATP- and GTP-binding Aptamers from Single RNA Sequences by Error-prone Replication and Selection

**Authors:** Falk Wachowius, Benjamin T. Porebski, Christopher M. Johnson & Philipp Holliger*

**Table of contents**

Supplementary Figures 1 - 14 p. 2 - 16

Supplementary Tables 1 – 3 p. 17 - 25

References p. 26

# Supplementary Figures

T5 GGATGAGCGACGCTGAAAAAAAAAAAAAAAAAAAAAAAAAAAAAAAAAAAAAAAAGTCTGGCGACTGCTC

| | | Abundance | |
|---|---|---|---|
| T5R0 | 1. | 37 | GGATGAGCGACGCTGAAAAATAGTCTGGCGACTGCTC |
| | 2. | 34 | GGATGAGCGACGCTGAAAGTCTGGCGACTGCTC |
| | 3. | 34 | GGATGAGCGACGCTGAAGTCTGGCGACTGCTC |
| | 4. | 28 | GGATGAGCGACGCTGAAAAGTCTGGCGACTGCTC |
| | 5. | 25 | GGATGAGCGACGCTGAGTCTGGCGACTGCTC |
| | 6. | 25 | GGATGAGCGACGCTGAAAATAGTCTGGCGACTGCTC |
| | 7. | 24 | GGATGAGCGACGCTGAAAAAATAGTCTGGCGACTGCTC |
| | 8. | 16 | GGATGAGCGACGCTGAAGGTCTGGCGACTGCTC |
| | 9. | 15 | GGATGAGCGACGCTGAAAGGTCTGGCGACTGCTC |
| | 10. | 15 | GGATGAGCGACGCTGGGTCTGGCGACTGCTC |
| T5R1 | 1. | 4 | GGATGAGCGACGCTGAAAAAAAAATAGTCTGGCGACTGCTC |
| | 2. | 3 | GGATGAGCGACGCTGAAAAAAAAAAATAGTCTGGCGACTGCTC |
| | 3. | 3 | GGATGAGCGACGCTGAAAAAAATAGTCTGGCGACTGCTC |
| | 4. | 2 | GGATGAGCGACGCTGAAAAAATAGTCTGGCGACTGCTC |
| | 5. | 2 | GGATGAGCGACGCTGAAAAAAAGGGTCTGGCGACTGCTC |
| | 6. | 2 | GGATGAGCGACGCTGAAAAAAAAACAGGTCTGGCGACTGCTC |
| | 7. | 2 | GGATGAGCGACGCTGAAAAACAGTCTGGCGACTGCTC |
| | 8. | 1 | GGATGAGCGACGCTGAAAATAAAAAGAAAATAAAAAAAAAGAGAAAAGAAAAAGTAGTCTGGCGACTGCTC |
| | 9. | 1 | GGATGAGCGACGCTGTAAAAAAAAAGAAAAAAAACAGAGAAAAAGAAGAAAAAAAGTCTGGCGACTGCTC |
| | 10. | 1 | GGATGAGCGACGCTGATAAATAAAAAAGAAAAAAAAAAGAAAAGATAAAAAGTAAAGGTCTGGCGACTGCTC |
| T5R2 | 1. | 2 | GGATGAGCGACGCTGATATTAAAAGAAAAAAAGAAAGAAGAAAGATGTAAAGGGTCTGGCGACTGCTC |
| | 2. | 2 | GGATGAGCGACGCTGAAAAAAAGAAAAAAGAAAAAGAAAAAAGAAAAAAAGAGAATATAAAGGGGTCTGGCGACTGCTC |
| | 3. | 2 | GGATGAGCGACGCTGAAAAGAAAAGAAAAAGAAAAAAAAGAGAAGTAGATATGGGTCTGGCGACTGCTC |
| | 4. | 2 | GGATGAGCGACGCTGAAAGAAAAAAAGAAAAAAGACAAAGGTCTAAGGTCTGGCGACTGCTC |
| | 5. | 2 | GGATGAGCGACGCTGATAAAAAAAGGAGAAAAAAAAGAGGAAAAGAAAAACCAGGGTCTGGCGACTGCTC |
| | 6. | 2 | GGATGAGCGACGCTGAAAAAAAAAGAATAAAAAAAGGAAAAAGGAAGAGAAAACATTAGTCTGGCGACTGCTC |
| | 7. | 1 | GGATGAGCGACGCTGAAAAAAAGAAAGAAAGAGAGAAAACAAAAAAAGAAAGAGGGTCTGGCGACTGCTC |
| | 8. | 1 | GGATGAGCGACGCTGGTAAAAAGAAATAGAAAAAAAACAATAAGAAAAAGAAGGGAAGGTCTGGCGACTGCTC |
| | 9. | 1 | GGATGAGCGACGCTGAAAAAAAAGATTAAAAAAGAAAAAAAGACGGATGGTCTGGCGACTGCTC |
| | 10. | 1 | GGATGAGCGACGCTGAAAAGAAAAATAAAGAGAGAAAAAATGAAAAGAATGGGGTCTGGCGACTGCTC |
| T5R3 | 1. | 2 | GGATGAGCGACGCTGAAAAAACAAAAAAAATAAAAAAAGAAAAAAGTCTGGCGTCTGGCGACTGCTC |
| | 2. | 2 | GGATGAGCGACGCTGGAATAAAAAAAGAAAGACAAGAAAAAAAGAAAAAGAGTCTGGTCTGGCGACTGCTC |
| | 3. | 2 | GGATGAGCGACGCTGAAAAAAAGAAAGAAAAAATAAAAAGGAAAAAAGAAAAGTCTAGTCTGGCGACTGCTC |
| | 4. | 2 | GGATGAGCGACGCTGAGAAGAAAGAGAAAGGAAAGAAAAAAAAAGACAATGGTCTGGCGACTGCTC |
| | 5. | 1 | GGATGAGCGACGCTGAAAAAAAAAGAAAAAAGAAAAAAAAAAGATTAGAAAAAGAAGGGGTCTGGCGACTGCTC |
| | 6. | 1 | GGATGAGCGACGCTGTTGGAAAATGAAAGAAAGGAAAGAAAAAAAAAGAACAGTCTGTCTGGCGACTGCTC |
| | 7. | 1 | GGATGAGCGACGCTGAAAAGAAAAAAAGAAAGGAAAGTGTCCGTCTGGCGACTGCTC |
| | 8. | 1 | GGATGAGCGACGCTGAGAATAAAAGAAAAGAAAGAAAAAAAGCAAAAAAAGAAGGGTCTGGCGACTGCTC |
| | 9. | 1 | GGATGAGCGACGCTGAAAGAGATAAACGAAGAAAAAAAAGAGAAAAAAGTCCGGCGTCTGGCGACTGCTC |
| | 10. | 1 | GGATGAGCGACGCTGAAAAATAAAAGGGAAAAGGAAGAAAAAAAAAATAGTCTGGCGGCTGGCGACTGCTC |
| T5R4 | 1. | 2 | GGATGAGCGACGCTGAAAATAAGAAAAAAAGAATAGAAAAGAAAAAAAAAGCCTGGTGTCTGGCGACTGCTC |
| | 2. | 2 | GGATGAGCGACGCTGAAAATAAGAAAAAAAGAATAGAAAAGAAAAAAAAAGCCTGGTGTCTGGCGACTGCTC |
| | 3. | 2 | GGATGAGCGACGCTGGAAGAAGAAGAAATAGAGGAAAAAAGAAAAAACGTCTGGGTCTGGCGCTGCTC |
| | 4. | 2 | GGATGAGCGACGCTGAAATAAAAAAAAATAAAAAAAAGAAAAAGAAAAGTCTGGCGTCTGGCGACTGCTC |
| | 5. | 2 | GGATGAGCGACGCTGAAAAAAGAAAAAAAAAGAAAAAAGAAAAAAAGAAAAAAAAGTCTGGTCTGGCGACTGCTC |
| | 6. | 2 | GGATGAGCGACGCTGAAAAAAAAAGAAAAAAAAGAAAGAAAAAGAGAAAAGGCCTGGTCTGGCGACTGCTC |
| | 7. | 1 | GGATGAGCGACGCTGAAAAAAAAAAGAAAAAATGAAAAAAAGGAAAAAAGTCTGGCGTCTGGCGACTGCTC |
| | 8. | 1 | GGATGAGCGACGCTGACAAAAAAAATCATAAAAAAAGAGAAAAACAAAAAAAAAGTCTGGCGTCTGGCGACTGCTC |
| | 9. | 1 | GGATGAGCGACGCTGGAAAAAGAAAAAGAAGAAAAGAAGATGAATATACGATGGTCTGGCGACTGCTC |
| | 10. | 1 | GGATGAGCGACGCTGAAAGAGAAAAAAAATTAAGTAGAAGAAAGTAAATAGAGTCTGGGTCTGGCGACTGCTC |
| T5R5 | 1. | 2 | GGATGAGCGACGCTGAGAAAAAGGAAAAAAGAAAAAAAGGAAGAAAAAAAAGTCTGGTCTGGCGACTGCTC |
| | 2. | 2 | GGATGAGCGACGCTGAGAAAAAAAAAATGAAAAAAGAGATCAATAAAGAAGTCCGGGTCTGGCGACTGCTC |
| | 3. | 1 | GGATGAGCGACGCTGAGACAAAGAAGAAAGCAAAAGAAAAAAAGGATAGTCCGGCAACTGGTCTGGCGACTGCTC |
| | 4. | 1 | GGATGAGCGACGCTGAAAGAGACAAAGAAAAAAAGAAAAATCAAGTAAAAGTCTGGCGTCTGGCGACTGCTC |
| | 5. | 1 | GGATGAGCGACGCTGAAAAAGAGGAAAAAGAAAGAAAAAAAGAAAGCGTCTGGTCTGGCGACTGCTC |
| | 6. | 1 | GGATGAGCGACGCTGAAGAAAAAAATAAACAAAAAGAAAGAAAAAGTCCGGCGTCTGGCGACTGCTC |
| | 7. | 1 | GGATGAGCGACGCTGAGAAAAAGGTAAAAAAGAGGAAAAAAAGGGAAAAGTCTGGCGTCTGGCGACTGCTC |
| | 8. | 1 | GGATGAGCGACGCTGAAAAAAAGAAAAGAAAGAAGAAAAAAAGGGGAAGCGTCTGGGTCTGGCGACTGCTC |
| | 9. | 1 | GGATGAGCGACGCTGATAAAAAAAAAATAAAAAAAAGAATGAAAACGAAAGAAGATGTGTCTGAGTCTGGCGACTGCTC |
| | 10. | 1 | GGATGAGCGACGCTGTAAAAAAAGAAAAGAAAGATAAAAAAAAAAAGAAAGCCGGGCCTGGCGACTGCTCTC |
| T5R6 | 1. | 2 | GGATGAGCGACGCTGAAAAAAAGGAAAAAAATGAAGAGAAAAAAAGAAGTGTCTAGTCTGGCGACTGCTC |
| | 2. | 1 | GGATGAGCGACGCTGATAATAAGAAGTAACAAAAACGAAAAAAAGAAGCCTGGGTCTGGCGACTGCTC |
| | 3. | 1 | GGATGAGCGACGCTGAAAGAGAAAAGAAAAGAAAAATGGAAAAAGAATAGAGAAAGGTCTGGTCTGGCGACTGCTC |
| | 4. | 1 | GGATGAGCGACGCTGAGGAAAGAAAGGAAAAAAGAATGAAACAGGGAAAGGTCTGGGCTGGCGACTGCTC |
| | 5. | 1 | GGATGAGCGACGCTGAAAAAAAAAAGAAGAAAAAAGAGGAGCAAACATGGTCTGGCGACTGCTC |
| | 6. | 1 | GGATGAGCGACGCTGAAAAAAAAAGAGAAAAAAAAAGTGAAAAAAGGAAAAAGCCTGGGTCTGGCGACTGCTC |
| | 7. | 1 | GGATGAGCGACGCTGAGAAAAAGAAAGAAAATAAAAAACGTAAAGAGAAAACCGGCGTCTGGCGACTGCTC |
| | 8. | 1 | GGATGAGCGACGCTGGAAAAATAAAGAAAAAGAAAGAGAATGAAAAAAAACCGTCTGGTCTGGCGACTGCTC |
| | 9. | 1 | GGATGAGCGACGCTGTAAAAGAAAAAGATAGAAAAAAAGAATAAAAAAAGCCTGGCGTCTGGCGACTGCTC |
| | 10. | 1 | GGATGAGCGACGCTGAAAGATAAAAAAAAAGAAAAGAATAAAAAAGAATGCCTGGGTCTGGCGACTGCTC |
| T5R7 | 1. | 9 | GGATGAGCGACGCTGGAAGGAAGAAAATGCAGAAAAAAAGAAAAAAATGTCTGGGTCTGGCGACTGCTC |
| | 2. | 4 | GGATGAGCGACGCTGGAAGGAAGAAAATGCAGAAAAAAAGAAGAAAATGTCTGGGTCTGGCGACTGCTC |
| | 3. | 2 | GGATGAGCGACGCTGGAAAGAAGAAAAGAAAAGAAAAGAAGAAAAGGAACCGTAGTCTGGCGACTGCTC |
| | 4. | 2 | GGATGAGCGACGCTGGAAGGAAGAAAATGCAGAAAAAAAGAAAGAAATGTCTGGGTCTGGCGACTGCTC |
| | 5. | 2 | GGATGAGCGACGCTGGAAGGAAGAAAATGCAGAAAAAAAGAAAAAAGTGTCTGGGTCTGGCGACTGCTC |
| | 6. | 2 | GGATGAGCGACGCTGGAAGGAAGAAAACGCAGAAAAAAAGAAAAAAATGTCTGGGTCTGGCGACTGCTC |
| | 7. | 2 | GGATGAGCGACGCTGGAAGGAAGAAAATGCAGAAAAGAAGAAAAAAATGTCTGGGTCTGGCGACTGCTC |
| | 8. | 2 | GGATGAGCGACGCTGGCAGGAAGAAAATGCAGAAAAAAAGAAAAAAATGTCTGGGTCTGGCGACTGCTC |
| | 9. | 2 | GGATGAGCGACGCTGGAAGGAAGAAAATGCAGAAGAAAAGAAAAAAAATGTCTGGGTCTGGCGACTGCTC |
| | 10. | 2 | GGATGAGCGACGCTGATCAAGAAAAATAAAGAGGATAAACGAGTATAAATGTGTGGCGTCTGGCGACTGCTC |
| T5R8 | 1. | 694 | GGATGAGCGACGCTGGAAGGAAGAAAATGCAGAAAAAAAGAAAAAAATGTCTGGGTCTGGCGACTGCTC |
| | 2. | 148 | GGATGAGCGACGCTGGAAGGAAGAAAATGCAGAAAAAAAGAAATAAATGTCTGGGTCTGGCGACTGCTC |
| | 3. | 122 | GGATGAGCGACGCTGGAAGGAAGAAAATGCAGAAAAAAAGAAAAGAATGTCTGGGTCTGGCGACTGCTC |
| | 4. | 81 | GGATGAGCGACGCTGGAAGGAAGAAAATGCAGAAAAAAAGAAAAAAACGTCTGGGTCTGGCGACTGCTC |
| | 5. | 75 | GGATGAGCGACGCTGGAAGGAAGAAAATGCAGAAAAAAAAGAAAAAAATGTCTGGGTCTGGCGACTGCT |
| | 6. | 73 | GGATGAGCGACGCTGGAAGGAAGAAAATGCAGAACAAAAGAAAAAAATGTCTGGGTCTGGCGACTGCTC |
| | 7. | 73 | GGATGAGCGACGCTGGAAGGAAGAAAATGCAGAGAAAAAGAAAAAAATGTCTGGGTCTGGCGACTGCTC |
| | 8. | 72 | GGATGAGCGACGCTGGAAGGAAGAAAATGCAGAAAAAAAGAAAAAATGTCTGGGTCTGGCGACTGCTC |
| | 9. | 69 | GGATGAGCGACGCTGGAAGGAAGAAAATGCAGAAAAAAAGAAGAAAATGTCTGGGTCTGGCGACTGCTC |
| | 10. | 67 | GGATGAGCGACGCTGGAAGGAAGAAAATGCAGAAAAAAAGAAAATAATGTCTGGGTCTGGCGACTGCTC |

***Supplementary Figure 1*. *Sequence emergence and abundance (T5 selection).*** *(top) T5 seed sequence is shown comprising central 39nt poly-A stretch (black) flanked by two 15nt conserved primer binding sequences (blue). (below) Ten most abundant sequences (1-10) for each selection round T5R0-T5R8 with abundance (nr of appearances in sequence pool) are shown. Early selection rounds (R0, R1) are dominated by truncated sequences. Appearance of non-canonical (5'-GGAAAAAAATG) (orange) in T5R6 and canonical ATP motif (5'-GGAAGAAAATG)(red) in T5R7 is highlighted.*

T7 GCACCGTGGACACAGAAAAAAAAAAAAAAAAAAAAAAAAAAAAAAAAAAAAAAAGACTGCCAGGTCGAG

| Round | Abundance | Sequence |
|---|---|---|
| T7R0 | 1-17 | GCACCGTGGACACAGAAAAAAAAGACTGCCAGGTCGAG |
| | 2-14 | GCACCGTGGACACAGAAAAAAAGACTGCCAGGTCGAG |
| | 3-13 | GCACCGTGGACACAGAAAAAAGACTGCCAGGTCGAG |
| | 4-13 | GCACCGTGGACACAGAAAAAAAAAGACTGCCAGGTCGAG |
| | 5-11 | GCACCGTGGACACAGAAAAAAAAAAGACTGCCAGGTCGAG |
| | 6-9 | GCACCGTGGACACAGAAAAGACTGCCAGGTCGAG |
| | 7-3 | GCACCGTGGACACAGAAAGACTGCCAGGTCGAG |
| | 8-3 | GCACCGTGGACACAGAAAAAGACTGCCAGGTCGAG |
| | 9-2 | GCACCGTGGACACAGTAAAAAAAAGACTGCCAGGTCGAG |
| | 10-2 | GCACCGTGGACACAGAAAAGAAAAAAAAGACTGCCAGGTCGAG |
| T7R1 | 1-26 | GCACCGTGGACACAGAAAAAAAAAGACTGCCAGGTCGAG |
| | 2-25 | GCACCGTGGACACAGACTGCCAGGTCGAG |
| | 3-18 | GCACCGTGGACACAGAAAAAAAGACTGCCAGGTCGAG |
| | 4-15 | GCACCGTGGACACAGAAAAAAAAAAGACTGCCAGGTCGAG |
| | 5-13 | GCACCGTGGACACAGAAAAAAAAGACTGCCAGGTCGAG |
| | 6-13 | GCACCGTGGACACAGGACTGCCAGGTCGAG |
| | 7-9 | GCACCGTGGACACAGAAAAAAGACTGCCAGGTCGAG |
| | 8-9 | GCACCGTGGACACAGAAGACTGCCAGGTCGAG |
| | 9-6 | GCACCGTGGACACAGAGACTGCCAGGTCGAG |
| | 10-5 | GCACCGTGGACACAGAAAAAGACTGCCAGGTCGAG |
| T7R12 | 1-2206 | GCACCGTGGACACAGAAAAGGAGATAGTGAATTAATGCGACTCACTTTTCCTCCGTGGACTGCCAGGTCGAG |
| | 2-1920 | GCACCGTGGACACAGAATAACAGGAGATAAAATAAAACTACGACTCTCTATACCGCTGTGGACTGCCAGGTCGAG |
| | 3-1501 | GCACCGTGGACACAGGAAAAAGAAGAGAGCGCAAAGAAAGGAAATGTCTCCCTGTGACTGCCAGGTCGAG |
| | 4-1395 | GCACCGTGGACACAGAAAAATAGAAAAGGGATAAAGCTACGTCTCACTATAGCTCTGTGGACTGCCAGGTCGAG |
| | 5-1183 | GCACCGTGGACACAGTGGATAAAGGAAGCATATGAAACTACAGCCCTCTATTTCCCTGTGGACTGCCAGGTCGAG |
| | 6-1165 | GCACCGTGGACACAGAAAAAGTAAATACGAATAAATACGACTTACTGACCTCTGTGGACTGCCAGGTCGAG |
| | 7-995 | GCACCGTGGACACAGAAAAAAGAAAGGTAAGATTAATACGACATACCTTCCACTGTGGACTGCCAGGTCGAG |
| | 8-955 | GCACCGTGGACACAGACAGAAAGGAAAAGCAAGAATAATAATAATCTACTGCCTGTGACTGCCAGGTCGAG |
| | 9-899 | GCACCGTGGACACAGAAGAGAGTGTATAAAAATTAATACGACACTAGACCTCTGTGGACTGCCAGGTCGAG |
| | 10-87 | GCACCGTGGACACAGGAAGGGACCAGAAAGAGTGGCTAAGAAAGAGTCCCCTGTGACTGCCAGGTCGAG |
| T7R16 | 1-725 | GCACCGTGGACACAGAAAAATATGAAGAAAAATGAAGCTGCGACTCTCCATAGCTCTGTGGACTGCCAGGTCGAG |
| | 2-660 | GCACCGTGGACACAGAAAAGGAGATAGTGAATTAATGCGACTCACTTTTCCTCCGTGGACTGCCAGGTCGAG |
| | 3-629 | GCACCGTGGACACAGTGGATAAAGGAAGCATATGAAACTACAGCCCTCTATTTCCCTGTGGACTGCCAGGTCGAG |
| | 4-572 | GCACCGTGGACACAGAAAAATATGAAGAAAAATGAAACTGCGACTCTCCATAGCTCTGTGGACTGCCAGGTCGAG |
| | 5-568 | GCACCGTGGACACAGGAAGGGACCAGAAAGAGTGGCTAAGAAAGAGTCCCCTGTGACTGCCAGGTCGAGGTCGAG |
| | 6-492 | GCACCGTGGACACAGAAAAATAAGAAAAAGAGATAAAGCTACGACTCTCTTCTTCACTGTGGACTGCCAGGTCGAG |
| | 7-453 | GCACCGTGGACACAGAATAACAGGAGATAAAATAAAACTACGACTCTCTATACCGCTGTGGACTGCCAGGTCGAG |
| | 8-417 | GCACCGTGGACACAGGAAAAAGAAGAGAGCGCAAAGAAAGGAAATGTCTCCCTGTGACTGCCAGGTCGAG |
| | 9-357 | GCACCGTGGACACAGGAAAAAGAAGAGAGCGCAAAGAGAGGAAATGTCTCCCTGTGACTGCCAGGTCGAG |
| | 10-343 | GCACCGTGGACACAGAATAACAGGAGATAAAATAAAGCTACGACTCTCTATACCGCTGTGGACTGCCAGGTCGAG |

***Supplementary Figure 2. Sequence emergence and abundance (T7 selection).*** *(top) T7 seed sequence is shown comprising central 39nt poly-A stretch (black) flanked by two 15nt conserved primer binding sequences (blue). (below) Ten most abundant sequences (1-10) for each selection rounds T7R0, T7R1, T7R12, T7R16 with abundance (nr of appearances in sequence pool) are shown. Early selection rounds (R0, R1) are dominated by truncated sequences.*

| | Abundance | Sequence |
|---|---|---|
| T8 | | GCCTGCAAGTGCGACAAAAAAAAAAAAAAAAAAAAAAAAAAAAAAAAAAAAAAAGAGACCACCGACGTG |
| T8R0 | 1-201 | GCCTGCAAGTGCGACAAAAAAAAAGAGACCACCGACGTG |
| | 2-194 | GCCTGCAAGTGCGACAAAAAAAAGAGACCACCGACGTG |
| | 3-124 | GCCTGCAAGTGCGACAAAAAAAGAGACCACCGACGTG |
| | 4-55 | GCCTGCAAGTGCGACAAAAAAAAAAGAGACCACCGACGTG |
| | 5-38 | GCCTGCAAGTGCGACAAAAAAGAGACCACCGACGTG |
| | 6-30 | GCCTGCAAGTGCGACAAAAAGAGACCACCGACGTG |
| | 7-17 | GCCTGCAAGTGCGACGAAAAAAAAAGAGACCACCGACGTG |
| | 8-16 | GCCTGCAAGTGCGACAAAAAAAAAAGAGACCACCGACGGG |
| | 9-14 | GCCTGCAAGTGCGACAAAAAAAAAGAAGAGACCACCGACGTG |
| | 10-13 | GCCTGCAAGTGCGACAAAAGAGACCACCGACGTG |
| T8R1 | 2-2 | GCCTGCAAGTGCGACAAAAAAAGAAAAAAAGATAAAAAAAAAGAAAAAAAAGAGACCACCGACGTG |
| | 3-2 | GCCTGCAAGTGCGACAAAAAAAAAGAAAAAATAAAAAAAGAAAAAAAAGAGACCACCGACGTG |
| | 4-1 | GCCTGCAAGTGCGACATAAAATAAAAAAATAATAGAAAAAGAAGAAAAAGAAAAGAGACCACCGACGTG |
| | 5-1 | GCCTGCAAGTGCGACAAAGAAAAAAAAAGAAAAAAGAAAAAAAAGAGACCACCGACGTG |
| | 6-1 | GCCTGCAAGTGCGACAAAAAAGGATAAAAAATAAAAAAGAAGAAGAAGAAAAAAGAGACCACCGACGTG |
| | 7-1 | GCCTGCAAGTGCGACAAAAAAAATAAAAAAAGAAAAAGAAAAAAAAATAAAAAGAGACCACCGACGTG |
| | 8-1 | GCCTGCAAGTGCGACAAAGCAAAAGGAAAAAAAGGAAAAAAAAATAAAAAGAAAAGAGACCACCGACGTG |
| | 9-1 | GCCTGCAAGTGCGACTAAAAAAAGAAAAGAAAATAAAGAAAATAAAAAGAAAAAGAGACCACCGACGTG |
| | 10-1 | GCCTGCAAGTGCGACAAAAAAAAAATAAAAAAAGAAAAAGAAAAAAAAAGAAAAGAAGAGACCACCGACGTG |
| | 11-1 | GCCTGCAAGTGCGACATAAGAAAAAACTAAAAAAAAAATAAAAAAAGAAAAAAGAGACCACCGACGTG |
| T8R12 | 1-4327 | GCCTGCAAGTGCGACCAAGGGAGGAAGAAAATGAAGAGGGAGACCTTCTACGGGAGACCACCGACGTG |
| | 2-837 | GCCTGCAAGTGCGACAGGAAGAAAATGAAGAACAAGAAAAGGAGGCCTCCGACATGAGACCACCGACGTG |
| | 3-456 | GCCTGCAAGTGCGACAGTGCAGGGAAGAAGAAAGAGTGGTAATTTTGCGTGTGAGACCACCGACGTG |
| | 4-443 | GCCTGCAAGTGCGACAAATGGGAAATAGCAAAAGAAAACAGAGACACCGACATGTTAGAGACCACCGACGTG |
| | 5-253 | GCCTGCAAGTGCGACTCCAAAGTAAAAACAGAGTGAGGGAAAAATGTTGCTATGGAGACCACCGACGTG |
| | 6-228 | GCCTGCAAGTGCGACCAAGGGAGGAAGAAGATGAAGAGGGAGACCTTCTACGGGAGACCACCGACGTG |
| | 7-199 | GCCTGCAAGTGCGACCAAGGGAGGAGGAAAATGAAGAGGGAGACCTTCTACGGGAGACCACCGACGTG |
| | 8-197 | GCCTGCAAGTGCGACCAAGGGAGGAAGAAAATGAAGAGGGAGACCTTCTGCGGGAGACCACCGACGTG |
| | 9-195 | GCCTGCAAGTGCGACCAAGGGAGGAAGAAAATGAAGAGGGAGACCTCCTACGGGAGACCACCGACGTG |
| | 10-193 | GCCTGCAAGTGCGACCTATAGAAAGAGAAATAAGAAGAAGGAGATCTCCTATGTGGAGACCACCGACGTG |
| T8R16 | 1-19321 | GCCTGCAAGTGCGACAAATGGGAAATAGCAAAAGAAAACAGAGACACCGACATGTTAGAGACCACCGACGTG |
| | 2-15939 | GCCTGCAAGTGCGACAGTGCAGGGAAGAAGAAAGAGTGGTAATTTTGCGTGTGAGACCACCGACGTG |
| | 3-12512 | GCCTGCAAGTGCGACGAATGGGAAATAGCAAAAGAAAACAGAGACACCGACATGTTAGAGACCACCGACGTG |
| | 4-9826 | GCCTGCAAGTGCGACAAATGGGAAATAGCAAAAGAAAACAGAGACACCGACATGCTAGAGACCACCGACGTG |
| | 5-8611 | GCCTGCAAGTGCGACTCACTGAATGGGAAGATGTGATGATGAGCCCTCCTACGTGAGACCACCGACGTG |
| | 6-5892 | GCCTGCAAGTGCGACAGATGGGAAATAGCAAAAGAAAACAGAGACACCGACATGTTAGAGACCACCGACGTG |
| | 7-4752 | GCCTGCAAGTGCGACAAGTGGGAAATAGCAAAAGAAAACAGAGACACCGACATGTTAGAGACCACCGACGTG |
| | 8-3475 | GCCTGCAAGTGCGACTCATATAAGGAGGGGAAAGGGGAGAGAGACCTCCTACGTGAGACCACCGACGTG |
| | 9-3471 | GCCTGCAAGTGCGACAAATGGGAGATAGCAAAAGAAAACAGAGACACCGACATGTTAGAGACCACCGACGTG |
| | 10-2893 | GCCTGCAAGTGCGACGAATGGGAAATAGCAAAAGAAAACAGAGACACCGACATGCTAGAGACCACCGACGTG |

***Supplementary Figure 3. Sequence emergence and abundance (T8 selection).*** *(top) T8 seed sequence is shown comprising central 39nt poly-A stretch (black) flanked by two 15nt conserved primer binding sequences (blue). (below) Ten most abundant sequences (1-10) for each selection rounds T8R0, T8R1, T8R12, T8R16 with abundance (nr of appearances in sequence pool) are shown. Early selection rounds (R0, R1) are dominated by truncated sequences. Appearance of the GTP binding core motif (5'-AATGGGAAATAGCAAAAGAAAACAGAGACACCGACATGTTA)(red) in T8R12 is highlighted.*

a) T4 GGCACTGCAGAGTCGTTTTTTTTTTTTTTTTTTTTTTTTTTTTTTTTTTTTTTTGACTCGAGCCAGAGC

| | Abundance | Sequence |
|---|---|---|
| T4R20 | 1-57 | GGCACTGCAGAGTCGTTATAGACTCACTATTATTTATCGCTCGTCTCCTCGTTGGACTCGAGCCAGAGC |
| | 2-52 | GGCACTGCAGAGTCGTAATTCCCTTTTTCTGCTCTACTTCTTTCTCTTTGGGTGGACTCGAGCCAGAGC |
| | 3-43 | GGCACTGCAGAGTCGTAATTCCCTTTTTCTGCTCTACTTCTTTTTCTTTGGGTGGACTCGAGCCAGAGC |
| | 4-38 | GGCACTGCAGAGTCGTTCTAGACTCACTATTTTTTATCGCTCGTCTCCTCGTTGGACTCGAGCCAGAGC |
| | 5-38 | GGCACTGCAGAGTCGTAATTCCCTTTTTCTGCTCTATTTCTTTCTCTTTGGGTGGACTCGAGCCAGAGC |
| | 6-33 | GGCACTGCAGAGTCGTAATTCCCTTTTTCTGCTCTACTTCTTTTTCCTTGGGTGGACTCGAGCCAGAGC |
| | 7-32 | GGCACTGCAGAGTCGTAATTCCCTTTTTCTGCTCTACTTCTTCTTCTTTGGGTGGACTCGAGCCAGAGC |
| | 8-30 | GGCACTGCAGAGTCGTAATTCCCTTTTTCGCTCTATTTCCTTTTCTTTGGGTGGACTCGAGCCAGAGC |
| | 9-29 | GGCACTGCAGAGTCGTAATTCCCTTTTTCTGCTCTATTTCTTTTTCTTTGGGTGGACTCGAGCCAGAGC |
| | 10-28 | GGCACTGCAGAGTCGTATAGACTCACTATTATTTATCGCTCGTCTCCTCGTTGGACTCGAGCCAGAGC |
| T4R24 | 1-364 | GGCACTGCAGAGTCGTCCTATGACTCATTTATTTTTATCGACTCGTTTCCCTGTGGACTCGAGCCAGAG |
| | 2-347 | GGCACTGCAGAGTCGTTCTATGACTCATTTACTTTTTTCGACTCGTTTCCCTGTGGACTCGAGCCAGAG |
| | 3-194 | GGCACTGCAGAGTCGTTATAGACTCACTATTATTTATCGCTCGTCTCCTCGTTGGACTCGAGCCAGAGC |
| | 4-186 | GGCACTGCAGAGTCGTTCTATGACTCATTTATTTTTTTCGACTCGTTTCCCTGTGGACTCGAGCCAGAGC |
| | 5-170 | GGCACTGCAGAGTCGTTCTATGACTCATTTATTTTTTTTCGACTCGTTTCCCTGTGGACTCGAGCCAGAG |
| | 6-159 | GGCACTGCAGAGTCGTATAGACTCACTATTATTTATCGCTCGTCTCCTCGTTGGACTCGAGCCAGAGC |
| | 7-139 | GGCACTGCAGAGTCGTTCTATGACTCATTTATTTTTATCGACTCGTTTCCCTGTGGACTCGAGCCAGAG |
| | 8-130 | GGCACTGCAGAGTCGTTATAGACTCACTATTATTTATCGCTCGTCTCCCCGTTGGACTCGAGCCAGAGC |
| | 9-129 | GGCACTGCAGAGTCGTTTTATGACTCATATATTTTATCGACTCGTTTCCGTGTGGACTCGAGCCAGAGC |
| | 10-121 | GGCACTGCAGAGTCGTTCTAGACTCACTATTTTTTATCGCTCGTCTCCTCGTTGGACTCGAGCCAGAGC |

T4R24 / 377

b) T6 GGACAGCGCGTGTAGTTTTTTTTTTTTTTTTTTTTTTTTTTTTTTTTTTTTTTTCTCCGTCGCTGCTAG

| | Abundance | Sequence |
|---|---|---|
| T6R20 | 1-15 | GGACAGCGCGTGTAGCCTTTTATTTTTCTCTCTCTGCTCAGGCTAGTACGCTCCTCCGTCGCTGCTAG |
| | 2-4 | GGACAGCGCGTGTAGCCTTTTATTTCTCTCTCTCTGCTCAGGCTAGTGCGCTCCTCCGTCGCTGCTAG |
| | 3-4 | GGACAGCGCGTGTAGCCTTTTATTTTTCTCTCTCTGCTCAGGCTAGTGCGCTCCTCCGTCGCTGCTAG |
| | 4-3 | GGACAGCGCGTGTAGCCTTTTATTTATCTCTCTCTGCTCAGGCTAGTACGCTCCTCCGTCGCTGCTAG |
| | 5-3 | GGACAGCGCGTGTAGCCTTTTATTTTTCCCTCTCTGCTCAGGCTAGTGCGCTCCTCCGTCGCTGCTAG |
| | 6-3 | GGACAGCGCGTGTAGCCTTTTATTTTCCTCTCTCTGCTCAGGCTAGTACGCTCCTCCGTCGCTGCTAG |
| | 7-3 | GGACAGCGCGTGTAGCCTTTTATTTTTCCCTCTCTGCTCAGGCTAGTACGCTCCTCCGTCGCTGCTAG |
| | 8-2 | GGACAGCGCGTGTAGACCTTCCCTCTTCTCCCTGCCTCCCTTTTTAGATCTACATGCTCCGTCGCTGCTAG |
| | 9-2 | GGACAGCGCGTGTAGCCTTTTATTTTTCTCTCTCAGCTCAGGCTAGTACGCTCCTCCGTCGCTGCTAG |
| | 10-2 | GGACAGCGCGTGTAGCCTTTTATTTTTCTCTCCCTGCTCAGGCTAGTACGCTCCTCCGTCGCTGCTAG |
| T6R24 | 1-3 | GGACAGCGCGTGTAGACCTTTTATTTTTCGGCTCTTCATAGGTCCTTCCCGCCTGCTCCGTCGCTGCTAG |
| | 2-3 | GGACAGCGCGTGTAGCCTTTTATTTTACTCTCTCTGCTCAGGCTAGTGCGCTCCTCCGTCGCTGCTAG |
| | 3-3 | GGACAGCGCGTGTAGCCTTTTATTTTCCTCTCTCTGCTCAGGCTAGTGCGCTCCTCCGTCGCTGCTAG |
| | 4-2 | GGACAGCGCGTGTAGACATCCCCAACTGCATCCTTCAGTCTCCATTCGCTGCTCCTCCGTCGCTGCTAG |
| | 5-2 | GGACAGCGCGTGTAGAGTTTATTTTTTGCCGCTTTTCTTCCTCTTCACGCTGCTCCTCCGTCGCTGCTAG |
| | 6-2 | GGACAGCGCGTGTAGACCCCACATACCAGCCTTTTTTCCTCACGTTGCCGCTCCTCCGTCGCTGCTAG |
| | 7-2 | GGACAGCGCGTGTAGCCTTACCCTTCCTCCTCTCTCCTCGCTCCGTCGCTACATGCTCCGTCGCTGCTAG |
| | 8-2 | GGACAGCGCGTGTAACCTAAACCCTCTTCCACACTCTCCGCTCCGCCGCTACATGCTCCGTCGCTGCTAG |
| | 9-1 | GGACAGCGCGTGTAGACCCCCTCCACTCCTCACTGCTCTTTTACCCTCTCTACATGCTCCGTCGCTGCTAG |
| | 10-1 | GGACAGCGCCTGTAGAGAATCCTGCTCCATCTCCTCATTTATACACACGCTCCGTCCTCCGTCGCTGCTAG |

T6R24 / 388

***Supplementary Figure 4. Sequence emergence and abundance (T4, T6 selections).*** *a) T4 seed sequence is shown comprising central 39nt poly-T stretch (black) flanked by two 15nt conserved primer binding sequences (blue). (below) Ten most abundant sequences (1-10) for each selection rounds T4R20, T4R24 with abundance (nr of appearances in sequence pool) are shown. a) T6 seed sequence is shown comprising central 39nt poly-T stretch (black) flanked by two 15nt conserved primer binding sequences (blue). (below) Ten most abundant sequences (1-10) for each selection rounds T6R20, T6R24 with abundance (nr of appearances in sequence pool) are shown.*

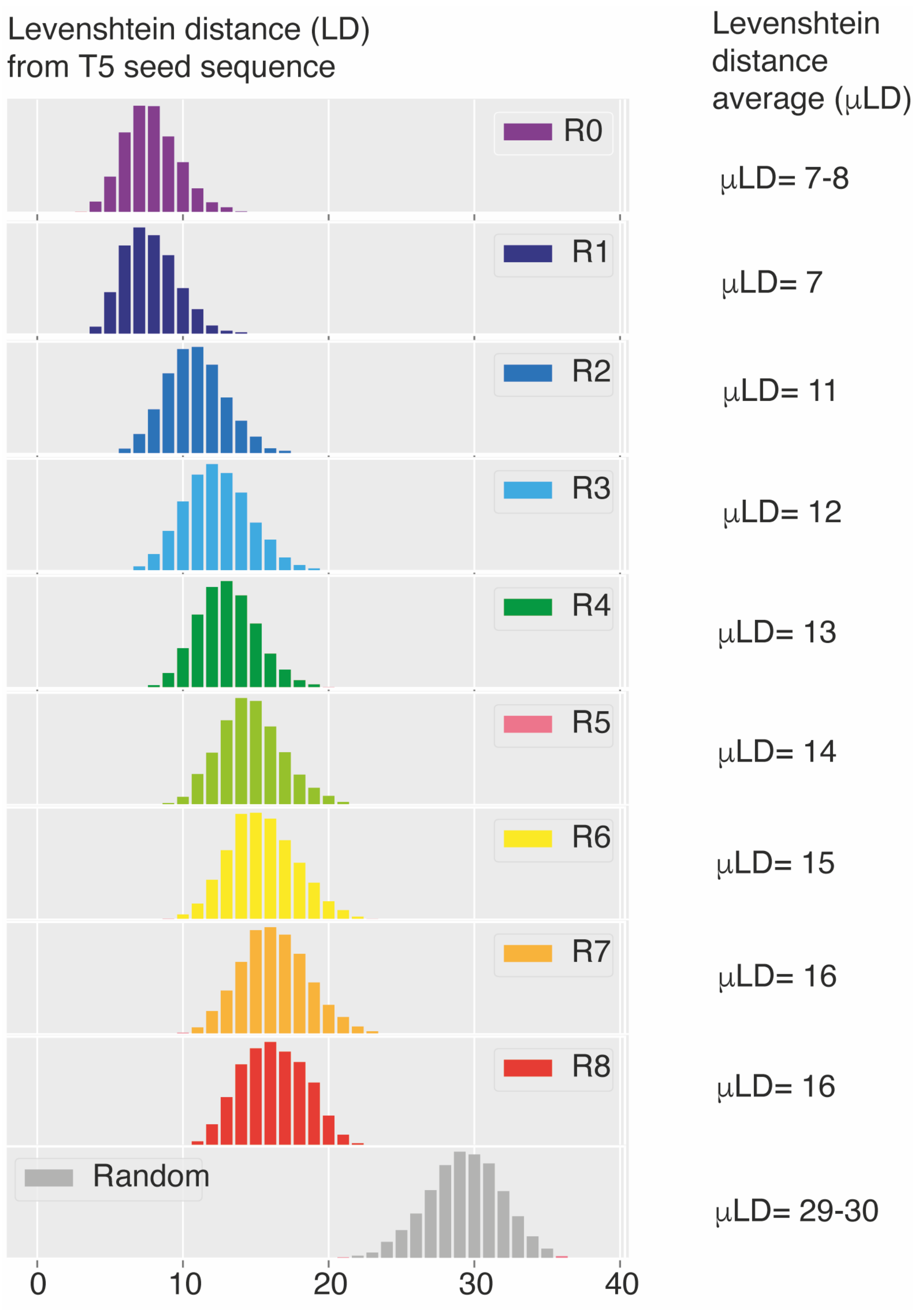


***Supplementary Figure 5. Levenshtein distance (HD).*** *Levenshtein distance (LD) distribution of T5 sequence pools at different points of the selection (R0-R8) with reference to a random sequence pool (dark grey). A LD of x between two sequences 1 and 2 means that x mutations are required to transform sequence 1 into sequence 2. Therefore, random sequences (grey) will be at a mutational (HD) distance distribution centered around 29 from any homopolymeric 39nt sequence. Thus T5 sequence pool (LDmax=16) never reaches the diversity of a random sequence pool.*

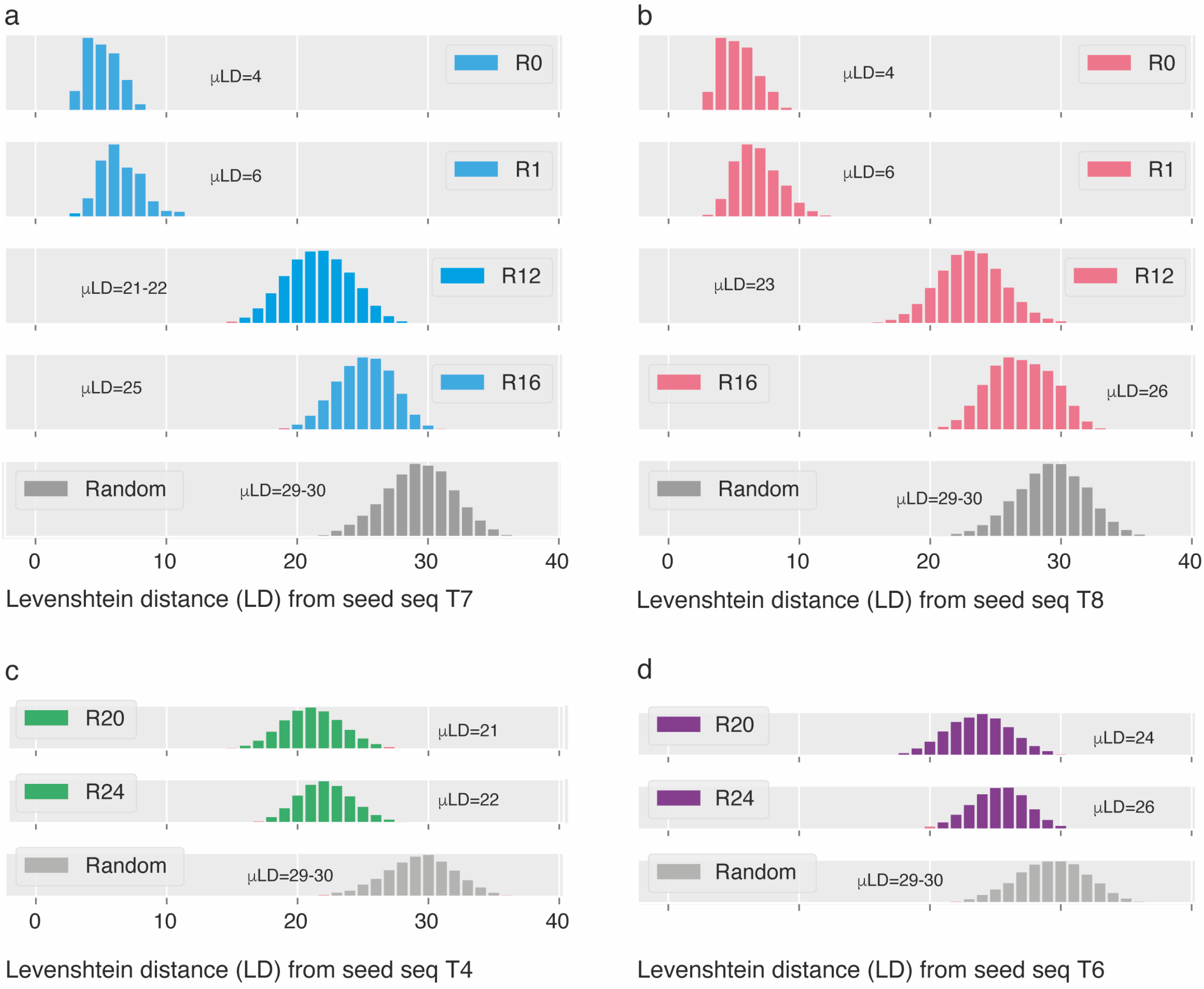


***Supplementary Figure 6*. *Levenshtein distance (HD).*** *Levenshtein distance (LD) distribution of a) T4, b) T6, c) T7, d) T8 sequence pools at different points of the selection (R0-R24) with reference to a random sequence pool (dark grey). A LD of x between two sequences 1 and 2 means that x mutations are required to transform sequence 1 into sequence 2. Therefore, random sequences (grey) will be at a mutational (HD) distance distribution centered around 29-30 from any homopolymeric 39nt sequence. Thus the T4, T6, T7, T8 sequence pools (*$LD_{max}$*= 22-26) never reach the diversity of a random sequence pool even after 24 rounds of error-prone replication and selection.*

a)

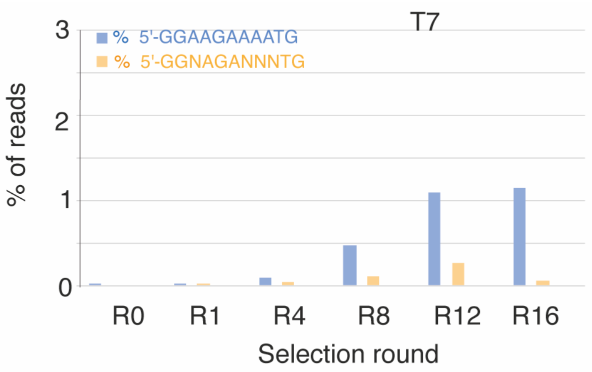


b)

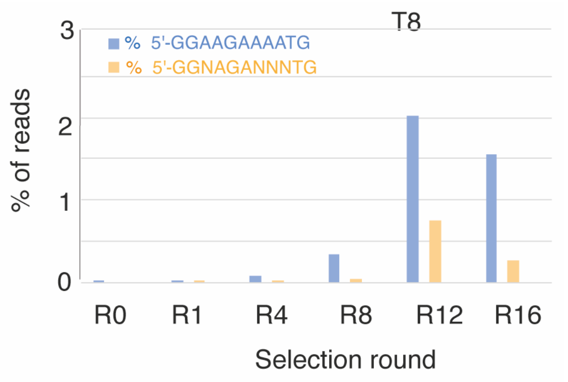


c)

| Sequence | Selection round | Number of sequence | N-NNN motif | A-AAA motif | N-NNN (%) | A-AAA (%) | N-NNN / A-AAA |
|---|---|---|---|---|---|---|---|
| T4 | R4 | 240569 | 0 | 0 | 0 | 0 | n.d. |
| | R8 | 317535 | 0 | 0 | 0 | 0 | n.d. |
| | R16 | 154726 | 0 | 0 | 0 | 0 | n.d. |
| | R20 | 1196046 | 4 | 0 | 0 | 0 | n.d. |
| | R24 | 590743 | 4 | 0 | 0.001 | 0 | n.d. |
| | | | | | | | |
| T6 | R4 | 55110 | 0 | 0 | 0 | 0 | n.d. |
| | R8 | 37816 | 0 | 0 | 0 | 0 | n.d. |
| | R16 | 12977 | 1 | 0 | 0.008 | 0 | n.d. |
| | R20 | 106422 | 6 | 0 | 0.006 | 0 | n.d. |
| | R24 | 95278 | 12 | 0 | 0.013 | 0 | n.d. |

***Supplementary Figure 7**. **Appearance of aptamer motif loops (see Figure 2a).** a, b) Ratio (%) from total unique sequences of the minimal (GGNAGA**NNN**TG (NNN) and near canonical GGAAGA**AAA**UG (AAA) ATP aptamer loop sequence motif (Canonical ATP aptamer motif (blue, cyan) and reverse motif (red, orange) for selection rounds (R0, R1, R4, R8, R12, R16) of the T7 and T8 selection experiments. c) Sporadic appearance of canonical aptamer motif loops in the T4 and T6 selection experiments. Reverse loops were not detected.*

a)

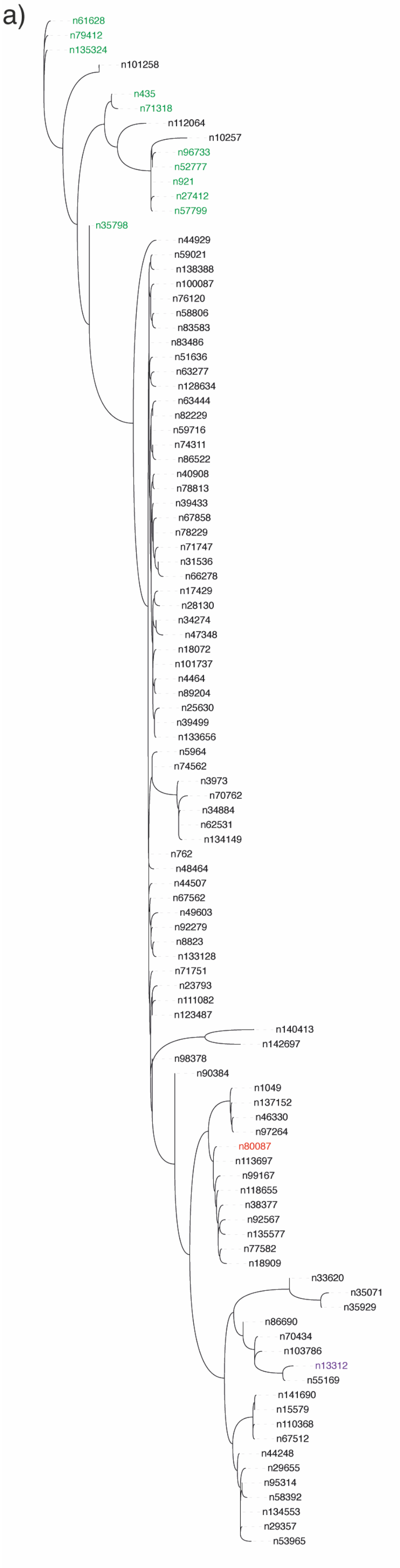


b)

```
435       1 GGATGAGCGACGCTGGA-GGAAGAAACTGCAGAGAGAAATAAGAAAAAGAAAGGTCTG-GTCTGGCGACTGCTC     72
762       1 GGATGAGCGACGCTGGATGGAAGAAAATGCAGAAAAAAAGAAAAAAATGTCTGG-----GTCTGGCGACTGCTC     69
921       1 GGATGAGCGACGCTGGA-GGAAGAAACTGCAGAGAGAAATAAGAAAAAAACGTCTG---GTCTGGCGACTGCTC     70
1049      1 GGATGAGCGACGCTGATGGGAAGAAAATGCAGAGGAAAAAAATAAAGTCTGGC------GTCTGGCGACTGCTC     68
3973      1 GGATGAGCGACGCTGATGGGAAGAAAATGCAGAGGAAAAGAAATAGAGTCTGGC-----GTCTGGCGACTGCTC     69
4464      1 GGATGAGCGACGCTGGACGGAAGAAAATGCAGACAAAGAGTCAAAAATGTCTGG-----GTCTGGCGACTGCTC     69
5964      1 GGATGAGCGACGCTGGACGGAAGAAAATGCAGAAGATAAGAAAAGAACGTCTGG-----GTCTGGCGACTGCTC     69
8823      1 GGATGAGCGACGCTGGATGGAAGAAAATGCAGAAAAAAAGGAATAAACGTCTGG-----GTCTGGCGACTGCTC     69
10257     1 GGATGAGCGACGCTGGA-GGAAGAAAATGGGGAAAAGAATAAGAATGAAGGCCTG--- GTCTGGCGACTGTTC     70
13312     1 GGATGAGCGACGCTGATGGGAAGAAAATGCAGAGGGAGAATATTAAAAAGTCTGGC---GTCTGGCGACTGCTC     71
17429     1 GGATGAGCGACGCTGGACGGAAGAAAATGCAGAAAAAAAGACATAGATGTCTGG-----GTCTGGCGACTGCTC     69
18072     1 GGATGAGCGACGCTGGGCGGAAGAAAATGCAGACAAAAGGACAAAAATGTCTGG-----GTCTGGCGACTGCTC     69
18909     1 GCATGAGCGACGCTGGACGGAAGAAAATGCAGACAAAAAGAAATAATGTCTGG------GTCTGGCGCCTGCTCA    69
25630     1 GGATGAGCGACGCTGGGCGGAAGAAAATGCAGACAAAAAGACAAGACGGTCTGG-----GTCTGGCGACTGCTC     69
27412     1 GGATGAGCGACGCTGGA-GGAAGAAACTGCAGAGAGAAATAAGAAAAGATCGTCTG---GTCTGGCGACTGCT      69
28130     1 GGATGAGCGACGCTGGACGGAAGAAAATGCAGAAAGAAAGACTATAATGTCTGG-----GTCTGGCGACTGCTC     69
29357     1 GGATGAGCGACGCTGGACGGAAGAAAATGCAGGAAAAAAAGAAAAAAATGTCTGG----GTCTGGCGACTGCT      69
29655     1 GGATGAGCGACGCTGGACGGAAGAAAATGCAGAAAAAGAAGAAAAAATAGTCTGG----GTCTGGCGACTGCT      69
31536     1 GGATGAGCGACGCTGGACGGAAGAAAATGCAGACGAAAAGCCATAAATGTCTGG-----GTCTGGCGACTGCTC     69
33620     1 GGATGAGCGACGCTGGATGGAAGAAAATGCAGAAATAAAGAAAAATGTCTGG-------GTCTGGCGACTGCTCAG  69
34274     1 GGATGAGCGACGCTGGACGGAAGAAAATGCAGACAATAAGACAAATATGTCTGG-----GTCTGGCGACTGCTC     69
34884     1 GGATGAGCGACGCTGATGGGAAGAAAATGCAGACGAAAAAAAATAAAGTCTGGC-----GTCTGGCGACTGCTC     69
35071     1 GGATGAGCGACGCTGATGGGAAGAAAATGCAGAGGAAAAAAAGTAGTCTGGC-------GTCTGGCGACTGCTCAG  69
35798     1 GGATGAGCGACGCTGGA-GGAAGAAACTGCAGAGAGAAATAAGAAAAAATCGTCTG----GTCTGGCGACTGCTC   69
35929     1 GGATGAGCGACGCTGATGGGAAGAAAATGCAGAGCGAAAAAAAAGGTCTGGC-------GTCTGGCGACTGCTCAG  69
38377     1 GGATGAGCGACGCTGGACGGAAGAAAATGCAGAAAAAAAAGTGAAAATGTCTGG------GTCTGGCGACTGCTCA  69
39433     1 GGATGAGCGACGCTGGACGGAAGAAAATGCAGACCAAAAGAAAAAAAATGTCTGG-----GTCTGGCGACTGCTC   69
39499     1 GGATGAGCGACGCTGGACGGAAGAAAATGCAGACAAAAAGACAAGAATGTCTGG-----GTCTGGCGACTGCTC     69
40908     1 GGATGAGCGACGCTGGACGGAAGAAAATGCAGACAAAAAGAAAGAAATGTCTGG-----GTCTGGAGACTGCTC     69
44248     1 GGATGAGCGACGCTGGACGGAAGAAAATGCGGACAAAAAAGAAAAAAATTTCTGG----GTCTGGCGACTGCT      69
44507     1 GGATGAGCGACGCTGGATGGAAGAAAATGCAGAAAAAAAGTAAAGAATGTCTGG-----GTCTGGCGACTGCTC     69
44929     1 GGATGAGCGACGCTGAGGCGGAAGAAAATGCAGAAAAAAAGAAAAAAAGTCTGG-----GTCTGGCGACTGCTC     69
46330     1 GGATGAGCGACGCTGATGGGAAGAAAATGCAGAGGAAATAAAAAATGTCTGGC------GTCTGGCGACTGCTCA   69
47348     1 GGATGAGCGACGCTGGACGGAAGAAAATGCAGAAAATAAGGGAAAGATGTCTGG-----GTCTGGCGACTGCTC     69
48464     1 GGATGAGCGACGCTGGGTGGAAGAAAATGCAGAAACAAAGAAAAAGATGTCTGG-----GTCTGGCGACTGCTC     69
49603     1 GGATGAGCGACGCTGGAAGGAAGAAAATGCAGAAAAAAAGTAATAAATGTCTGGT-----TTTGGCGACTGCTC    69
51636     1 GGATGAGCGACGCTGGACGGAAGAAAATGCAGAAAAAAGGCAAAAAATGTCTGG-----GTCTGGCGACTGCTC     69
52777     1 GGATGAGCGACGCTGGA-GGAAGAAACTGCAGAGAGAAATGAGAAAAAAACGTCTG---GTCTGGCGACTGCT      69
53965     1 GGATGAGCGACGCTGGACGGAAGAAAATGCAGAAAATTTGAAAAAGAATGTCTGG----GTCTGGCGACTGCT      69
55169     1 GGATGAGCGACGCTGATGGGAAGAAAATGCAGGGGAAAAAAAAAAAGAAGTCTGGC---GTCTGGCGACTGC      69
57799     1 GGATGAGCGACGCTGGA-GGAAGAAACTGCAGAGAGATATAAGAAAAAAACGTCTG---GTCTGGCGACTGCT      69
58392     1 GGATGAGCGACGCTGGGTGGAAGAAAATGCAGAAAAAAAGAAAAAAAATGTCTGG----GTCTGGCGACTGCT      69
58806     1 GGATGAGCGACGCTGGACGGAAGAAAATGCAGATAAAGAGAAGAAAATGTCTGG-----GTCTGGCGACTGCTC     69
59021     1 GGATGAGCGACGCTGGACGGAAGAAAATGCAGAAAAAAAGAAAATAGTGTCTGG-----GTCTGGCGACTGCTC     69
59716     1 GGATGAGCGACGCTGGACGGAAGAAAATGCAGAGAAAAAGAAAAAAATGTCTGG-----GTCTGGCGACTGCTC     69
61628     1 GGATGAGCGACGCTGGA-GGAAGAAACTGCAGAGAGAAATAAAAAAAGAAAGGTCTG--GTCTGGCGACTGC      69
62531     1 GGATGAGCGACGCTGATGGGAAGAAAATGCAGAGGAAAAAAAATAAAGTCTGGC-----GTCTGGCGACTGCTC     69
63277     1 GGATGAGCGACGCTGGACGGAAGAAAATGCAGAAAAAATGAGAAAAATGTCTGG-----GTCTGGCGACTACTC     69
63444     1 GGATGAGCGACGCTGGACGGAAGAAAATGCAGAATAAAAGAAAAATATGTCTGG-----GTCTGGCGACTGCTC     69
66278     1 GGATGAGCGACGCTGGACGGAAGAAAATGCAGAGTAAAAGCGATAAATGTCTGG-----GTCTGGCGACTGCTC     69
67512     1 GGATGAGCGACGCTGATGGGAAGAAAATGCAGAGGAATAAAAAATAAAGTCTGGC----GTCTGGCGACTGCT      69
67562     1 GGATGAGCGACGCTGGATGGAAGAAAATGCAGAAAAAAAGAAAGAAATGTCTGG-----GTCTGGCGACTGCTC     69
67858     1 GGATGAGCGACGCTGGACGGAAGAAAATGCAGACGATGAGAAAAAAATGTCTGG-----GTCTGGCGACTGCTC     69
70434     1 GGATGAGCGACGCTGGACGGAAGAAAATGCAGACAAAAAAAGAAGAAAATGTCTGG---GTCTGGCGACTGC      69
70762     1 GGATGAGCGACGCTGATGGGAAGAAAATGCAGAGGTAAATACAAAATGTCTGGC-----GTCTGGCGACTGCTC     69
71318     1 GGATGAGCGACGCTGGA-GGAAGAAACTGCAGAGAGAAATAAGAAAAGAATATGTCTG-GTCTGGCGACTG       69
71747     1 GGATGAGCGACGCTGGACGGAAGAAAATGCAGACGAAAAGCATAAGATGTCTGG-----GTCTGGCGACTGCTC     69
71751     1 GGATGAGCGACGCTGGATGGAAGAAAATGCAGACAAAATGAAAAAAATGTCTGG-----GTCTGGCGACTGCTC     69
74311     1 GGATGAGCGACGCTGGACGGAAGAAAATGCAGAGAAGAAGAAAAAAATGTCTGG-----GTCTGGCGACTGCTC     69
74562     1 GGATGAGCGACGCTGGATGGAAGAAAATGCAGAAGAAAAGAAAAAAACGTCTGG-----GTCTGGCGACTGCTC     69
76120     1 GGATGAGCGACGCTGGACGGAAGAAAATGCAGAAAAAGAGAAAAAAATGTCTGG-----GTCTGGCGACTGCTC     69
77582     1 GGATGAGCGACGCTGGACGGAAGAAAATGCAGACAAAAGGCAAAAATGTCTGG------GTCTGGCGACTGCTCA   69
78229     1 GGATGAGCGACGCTGGACGGAAGAAAATGCAGACGAAAAGAAAAAAATGTCTGG-----GTCTGGCGACTGCTC     69
78813     1 GGATGAGCGACGCTGGACGGAAGAAAATGCAGACAAAAAGAAAGGAATGTCTGG-----GTCTGGCGACTGCTC     69
79412     1 GGATGAGCGACGCTGGA-GGAAGAAACTGCAGAGAGAAAAAAGAAAAAAATCGTCTG--GTCTGGCGACTGC      69
80087     1 GGATGAGCGACGCTGGAAGGAAGAAAATGCAGAAAAAAAGAAAAAAAGTCTGG------TTCTGGCGACTGCTCA   69
82229     1 GGATGAGCGACGCTGGACGGAAGAAAATGCAGAGAAAAAGAAAAATATGTCTGG-----GTCTGGCGACTGCTC     69
83486     1 GGATGAGCGACGCTGGACGGAAGAAAATGCAGAAAAAAAGAAAAAAATGTCTGG-----GTCTGGCGACTGCTC     69
83583     1 GGATGAGCGTCGCTGGACGGAAGAAAATGCAGAAAAATAGAAGAAAATGTCTGG-----GTCTGGCGACTGCTC     69
86522     1 GGATGAGCGACGCTGGACGGAAGAAAATGCAGAGAAGAGGAAAAAAATGTCTGG-----GTCTGGCAACTGCTC     69
86690     1 GGATGAGCGACGCTGGACGGAAGAAAATGCAGACAAAAAAGAAAAAAAGTGTCTGG---GTCTGGCGATGCT      69
89204     1 GGATGAGCGACGCTGGACGGAAGAAAATGCAGACAAAAAGTCAAAAACGTCTGG-----GTCTGGCGACTGCTC     69
90384     1 GGATGAGCGACGCTGGACGGAAGAAAATGCAGAGAGAAAGAGACAAATGTCTGG-----GTCTGGC-ACTGCTCA   69
92279     1 GGATGAGCGACGCTGGATGGAAGAAAATGCAGAAAAATAGAAATAAATGTCTGG-----GTCTGGCGACTGCTC     69
92567     1 GGATGAGCGACGCTGGAAGGAAGAAAATGCAGAAAAAAAGAAAACAATGTCTGGT------TCTGGCGACTGCTCA  69
95314     1 GGATGAGCGACGCTGGATGGAAGAAAATGCAGAAAAAAAAAGAAAAAAATGTCTGG----GTCTGGCGACTGCT    69
96733     1 GGATGAGCGACGCTGGA-GGAAGAAACTGCAGAGGGAAATGAGAAAAAGACGTCTG---GTCTGGCGACTGCT      69
97264     1 GGATGAGCGACGCTGATGGGAAGAAAATGCAGAGGAAAAATAAAATGTCTGGC------GTCTGGCGACTGCTCA   69
98378     1 GGATGAGCGACGCTGGATGGAAGAAAATGCAGAGAAAAAGAGAAAAATGTCTGG-----GTCTGGCGACTGCTC    69
99167     1 GGATGAGCGACGCTGGATGGAAGAAAATGCAGAAGAAAAGAAAGGATGTCTGG------GTCTGGCGACTGCTCA  69
100087    1 GGATGAGCGACGCTGGATGGAAGAAAATGCAGAAAAAGAGAAAAAAGTGTCTGG-----GTCTGGCGACTGCTC    69
101258    1 GGATGAGCGACGCTGGA-GGAAGAAAATGCAGAAAAAAAAGAAAAAAATGTCTG-------GTCTGGCGACTGCTCAGA 69
101737    1 GGATGAGCGACGCTGGACGGAAGAAAATGCAGACAAAAAGACAAAAATGTCTGG-----GTCTGGCGACTGCTC    69
103786    1 GGATGAGCGACGCTGGATGGAAGAAAATGCAGAAAGAAAGAAAAAAAAAATGTCTGG---GTCTGGCGACTGC    69
110368    1 GGATGAGCGACGCTGATGGGAAGAAAATGCAGAGGAGAAAAAAAATAAAGTCTGGC----GTCTGGCGACTGCT   69
111082    1 GGATGAGCGACGCTGGGTGGAAGAAAATGCAGAGCAAAGGAAAAAAATGTCTGG-----GTCTGGCGACTGCTC    69
112064    1 GGATGAGCGACGCTGGA-GGAAGAAAATGCAGAGAAAAAGGAAAAAATGTCTG------GTCTGGCGACTGCTCAG 69
113697    1 GGATGAGCGACGCTGGATGGAAGAAAATGCAGAAAAAAAGAAAAAAAGTCTGG------GTCTGGCGACTGCTCA  69
118655    1 GGATGAGCGACGCTGGATGGAAGAAAATGCAGAGAAAAGGAAAAAATGTCTGG------GTCTGGCGACTGCTCA  69
123487    1 GGATGAGCGACGCTGGATGGAAGAAAATGCAGAGAAAAGGAAAAAAATGTCTGG-----GTCTGGCGACTGCTC    69
128634    1 GGATGAGCGACGCTGGACGGAAGAAAATGCAGAAAAGATGAAAACAATATCTGG-----GTCTGGCGACTGCTC    69
133128    1 GGATGAGCGACGCTGGATGGAAGAAAATGCAGGAAAGAAGAAATAAACGTCTGG-----GTCTGGCGACTGCTC    69
133656    1 GGATGAGCGACGCTGGACGGAAGAAAATGCAGACATAAAGACAAGAATGTCTGG-----GTCTGGCGACTGCTC    69
134149    1 GGATGAGCGACGCTGATGGGAAGAAACTGCAGAGGAAAAAAAATATAGTCTGGC-----GTCTGGCGACTGCTC    69
134553    1 GGATGAGCGACGCTGGACGGAAGAAAATGCAGAAAAAAAAAGAAAAAAATGTCTGG----GTCTGGCGACTGCT   69
135324    1 GGATGAGCGACGCTGGA-GGAAGAAACTGCAGAGAGAAATAAGAAGAAAACTGTCTG--GTCTGGCGACTGC     69
135577    1 GGATGAGCGACGCTGGACGGAAGAAAATGCAGAATGAAGAAAAAAGTGTCTGG------GTCTGGCGACTGCTCA  69
137152    1 GGATGAGCGACGCTGATGGGAAGAAAATGCAGAGGAAAAAAAAAAACGTCTGGC------GTCTGGCGACTGCTCA 69
138388    1 GGATGAGCGACGCTGGACGGAAGAAAATGCGGAACAAAAGAAAAAAGTGTCTGG-----GTCTGGCGACTGCTC    69
140413    1 GGATGAGCGACGCTGATGGGAAGAAATTGCAGAGGAGAAAAAAAGTCTGGC--------GTCTGGCGACTGCTCAGA 69
142697    1 GGATGAGCGACGCTGGACGGAAGAAAATGCAGAGAAAAAACATAAACAAAT--------GTCTGGGTCTGGCGACTG 69
```

***Supplementary Figure 8. Aptamer motifs.*** *a) Linear phylogenetic tree of b) predicted ATP aptamer motifs in T5R8 sequence pool (see Figure 2). Primer sequences (grey), aptamer loop and G-bulge (blue).*

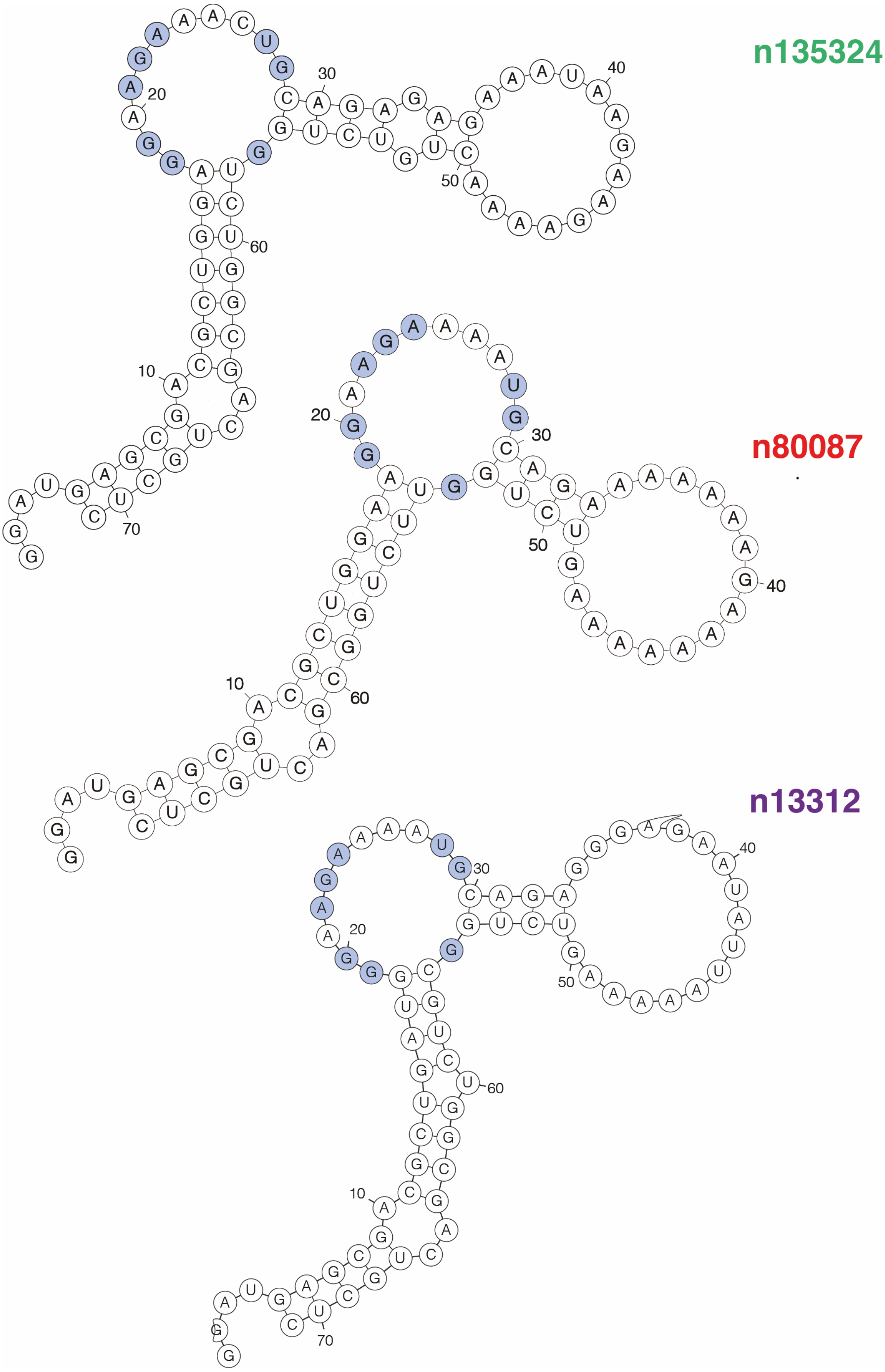


***Supplementary Figure 9*. *Aptamer motifs.*** *Predicted secondary structure* (RNA fold[16]) *of ATP aptamer motifs in T5R8 sequence pool (see Fig. 2, Supplementary Figure 8) of perfect match (n135324) and imperfect match (n13312, n80087) hits. Note the canonical ATP aptamer loop / G-bulge motif appears in all, with variations confined to the stem regions.*

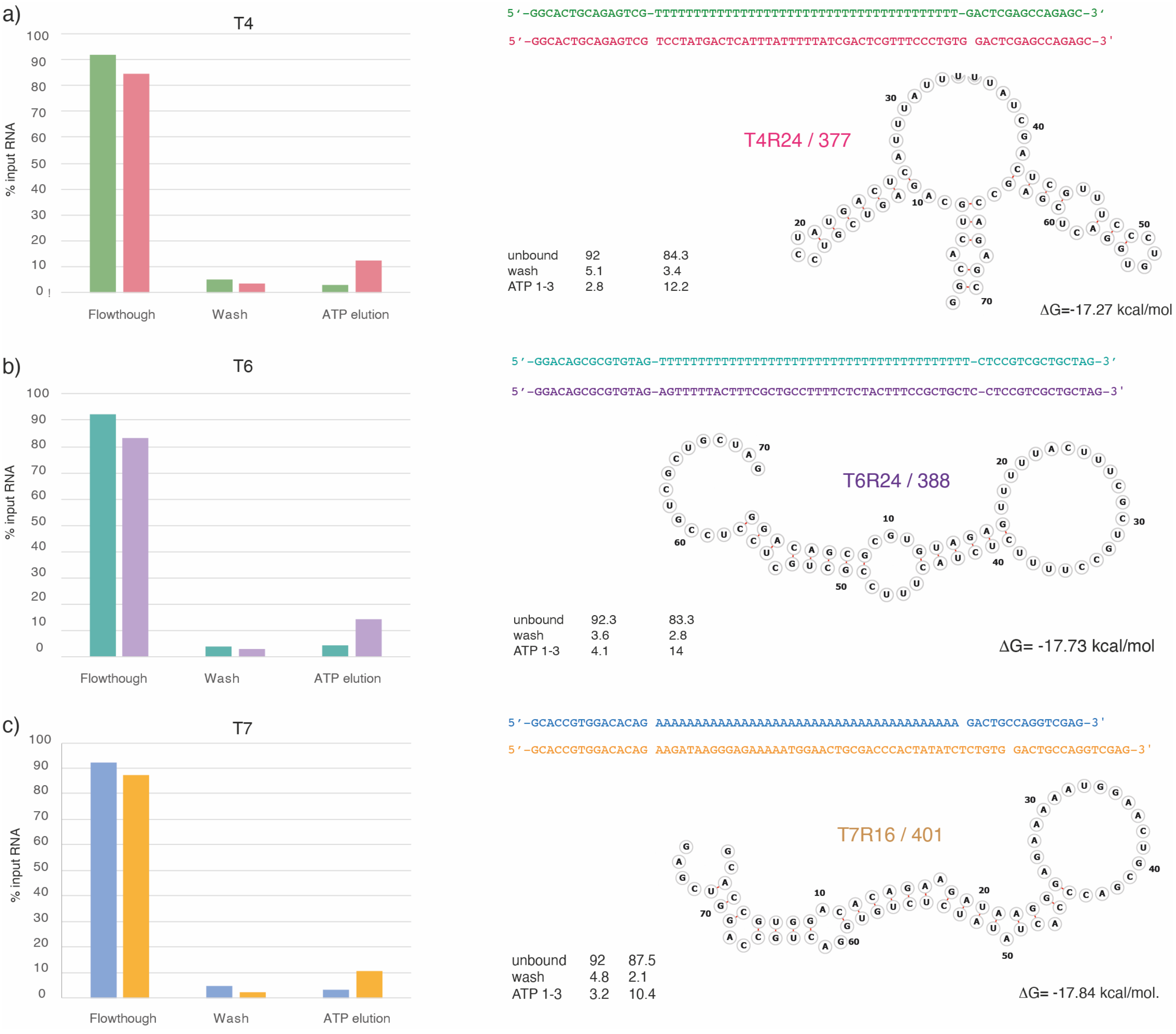


***Supplementary Figure 10*. *Putative ATP aptamer motifs.*** *(Left panel) ATP-sepharose column elution of a) T4, b) T6, c) T7, d) T8 seed sequences and the selected putative ATP aptamers a) T4R24/377, b) T6R24/388, c) T7R16/401. (Right panel) Sequence, predicted secondary structures and stabilities (ΔG)(as judged by* RNA fold[16]*) of putative aptamers.*

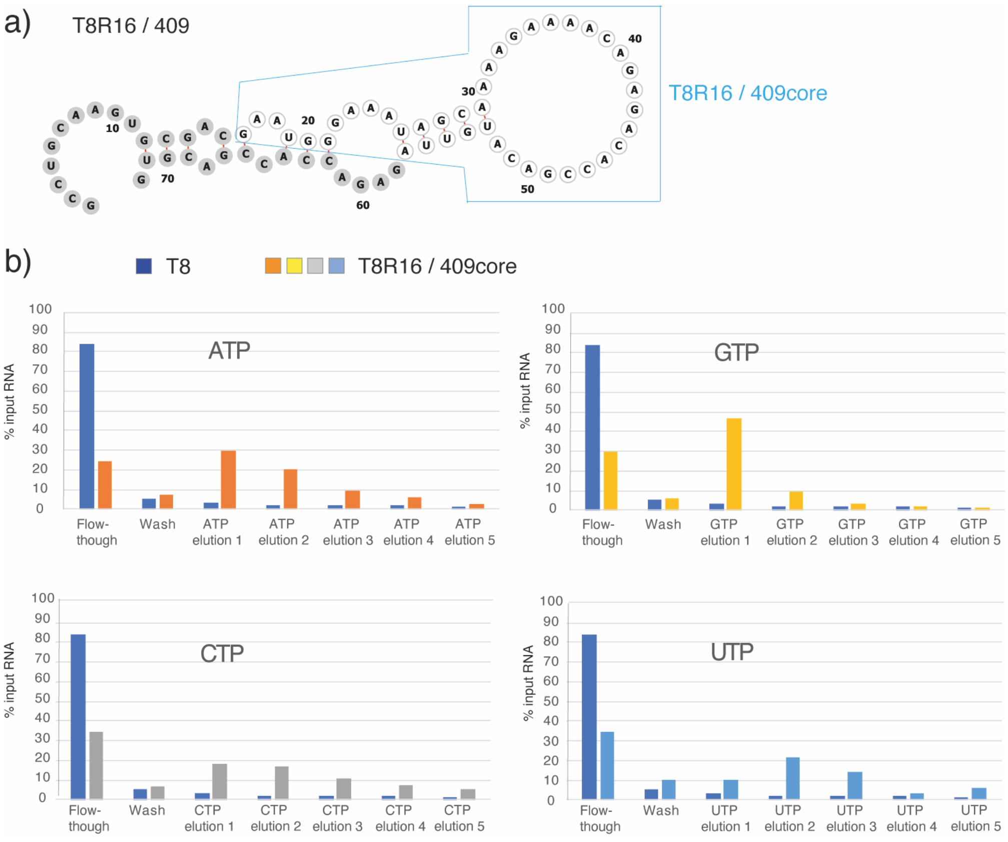


***Supplementary Figure 11. Putative nucleotide binding aptamer T8R16/409.** a) Predicted secondary structure (*RNA fold[16]*) of T8R16/409 aptamer with conserved primer sequences (grey) and core domain (T8R16/409core) (cyan box).b) ATP-agarose column elution of T8 seed sequence (blue) and T8R16/409core with ATP (orange), GTP (yellow), CTP (grey) and UTP (cyan) (see Supplementary Table 3). Note that T8R16/409core is eluted by all 4 nucleotides, but most efficiently by GTP.*

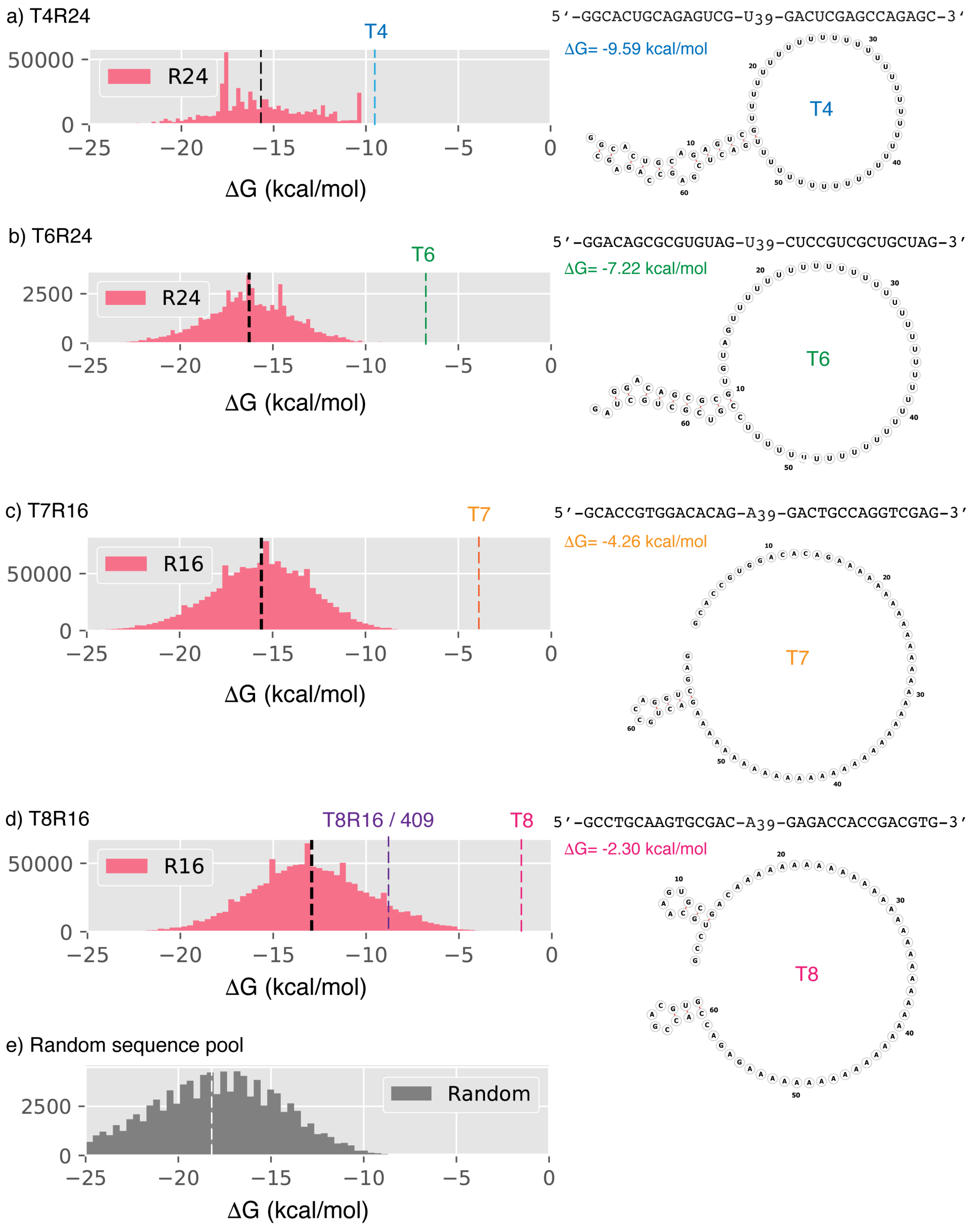


***Supplementary Figure 12. RNA folding.*** *(Left panel) RNA folding energy distributions for a) T4R24, b) T6R24, c) T7R16, d) R8R16, e) random sequence pools (median: black ((e) white)) dotted line. (right panel) Sequence, predicted secondary structure and folding energy (ΔG (cyan dotted line)) (*RNA fold[16]*) of T4, T6, T7, T8 seed sequences.*

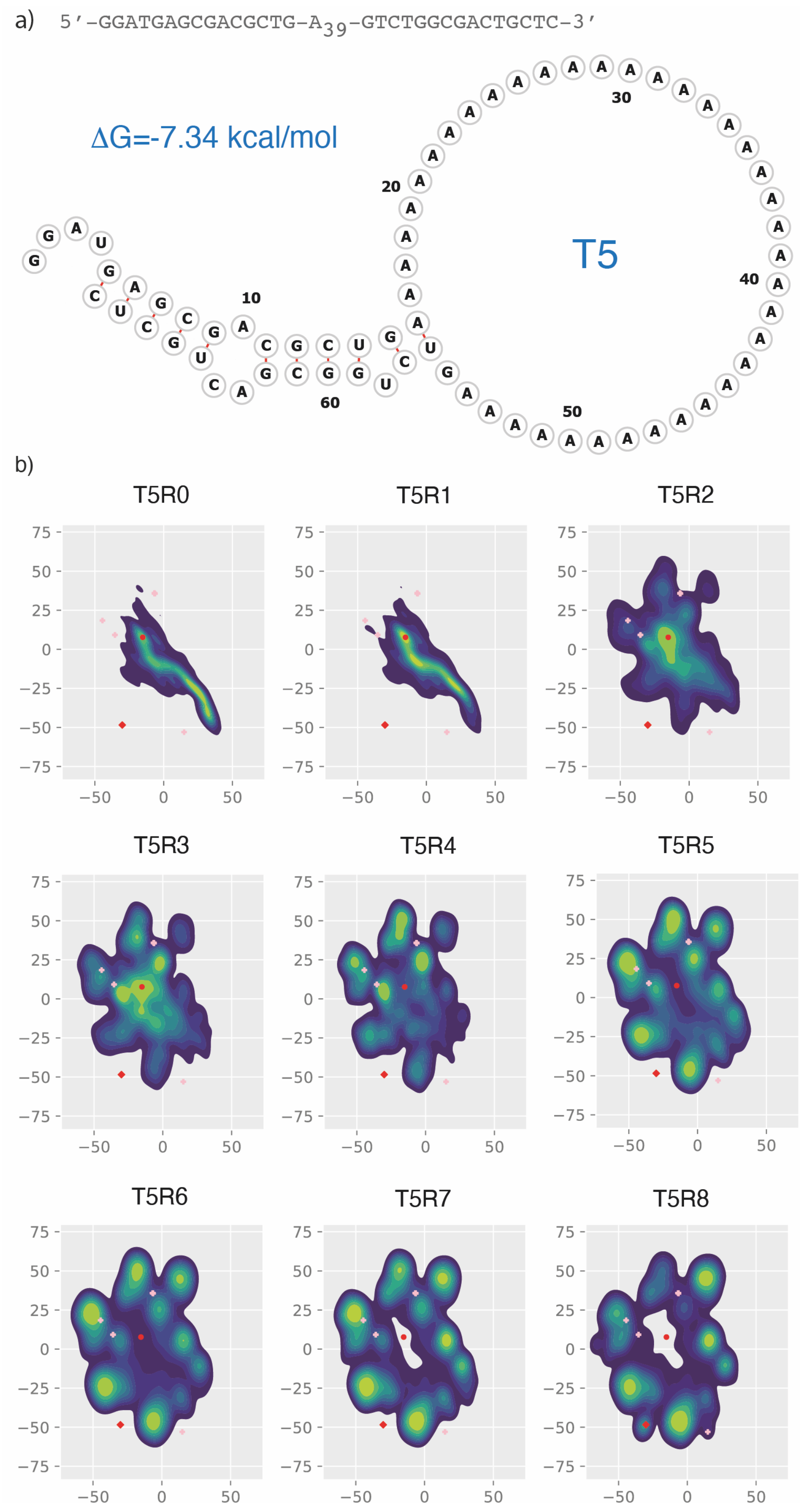


***Supplementary Figure 13**. **Sequence diversity in T5 selection.** a)* Sequence, predicted secondary structure and folding energy (ΔG) (RNA fold[16]) of T5 seed sequence. b) *Sequence space evolution as shown by t-stochastic neighbour embedding (tSNE[22])* as a two-dimensional projection visualisation *across the whole T5 selection trajectory for T5R0-T5R8 (T5 seed (red dot), T5R8/359 aptamer (red diamond), ATP aptamer motif perfect hits (Figure 2, pink crosses).*

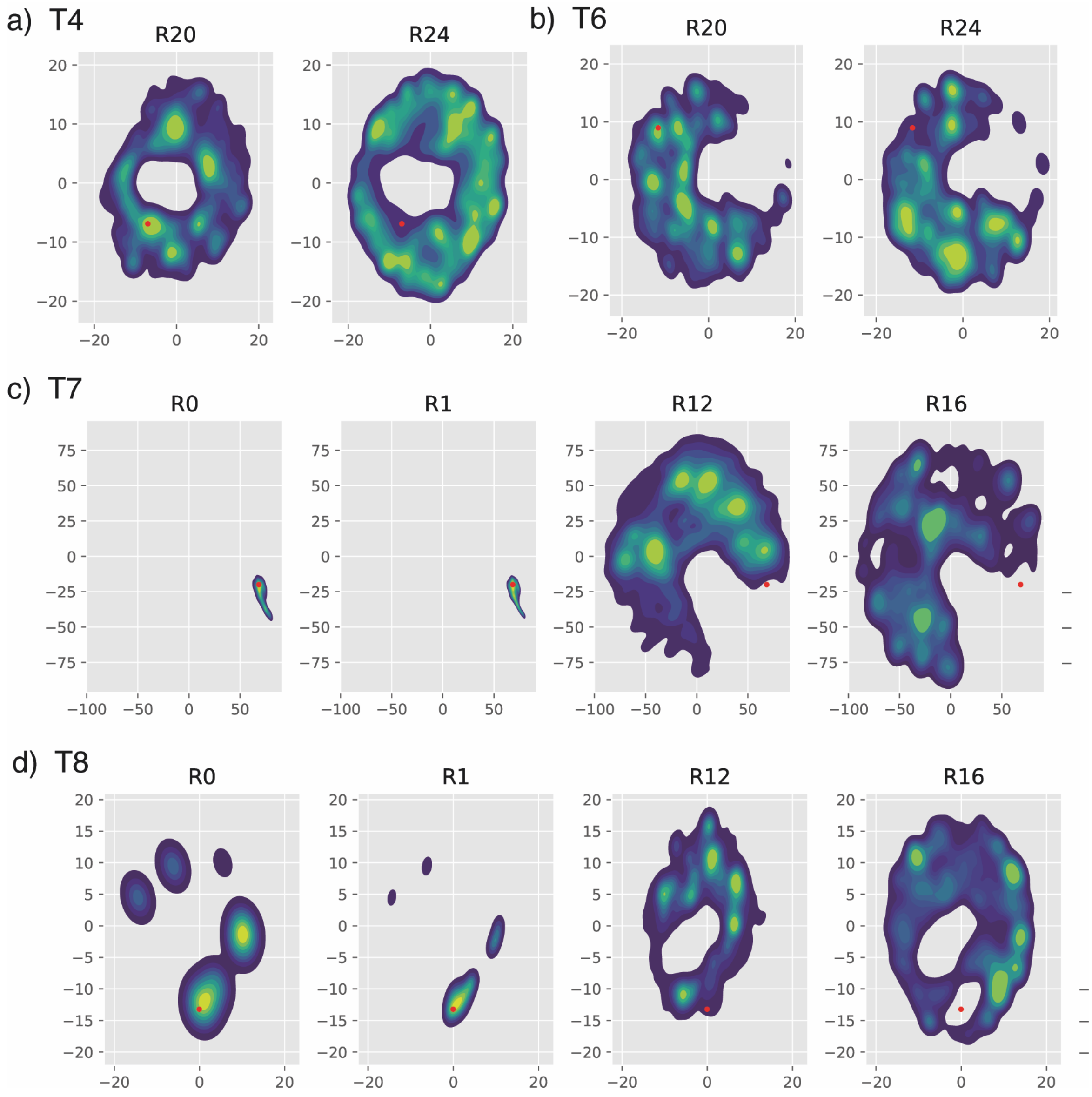


***Supplementary Figure 14***. ***Sequence diversity in T4, T6, T7 and T8 selections.*** Sequence space evolution as shown by t-stochastic neighbour embedding (*tSNE*[22]) as a two-dimensional projection visualisation *of a), T4, b) T6, c) T7, d) T8 at different points of the selection (R0-R24). T4, T6,T7,T8 seed sequences shown as red dot.*

**Supplementary Tables**

**Supplementary Table 1: Oligonucleotide sequences.**

The primer binding sites of the starting sequences are underlined. RNA sequences are shown as DNA templates for PCR. Sequencing barcodes shown in colour.

| Name | Function | Sequence |
|---|---|---|
| T4 | Starting sequence | 5'-GGCACTGCAGAGTCG TTTTTTTTTTTTTTTTTTTTTTTTTTTTTTTTTTTTTTTT GACTCGAGCCAGAGC-3' |
| T6 | Starting sequence | 5'-GGACAGCGCGTGTAG TTTTTTTTTTTTTTTTTTTTTTTTTTTTTTTTTTTTTTTT CTCCGTCGCTGCTAG-3' |
| T5 | Starting sequence | 5'-GGATGAGCGACGCTGAAAAAAAAAAAAAAAAAAAA AAAAAAAAAAAAAAAAAAA GTCTGGCGACTGCTC-3' |
| T7 | Starting sequence | 5'-GCACCGTGGACACAG AAAAAAAAAAAAAAAAAAAAAAAAAAAAAAAAAAAAAAAA GACTGCCAGGTCGAG-3' |
| T8 | Starting sequence | 5'-GCCTGCAAGTGCGAC AAAAAAAAAAAAAAAAAAAAAAAAAAAAAAAAAAAAAAAA GAGACCACCGACGTG-3' |
| T4 forward primer | Primer | 5'-GATCGATCTCGCCCGCGAAATTAATACGACTCACTATA GGCACTGCAGAGTCG-3' |
| T4 reverse primer | Primer | 5'-GCTCTGGCTCGAGTC-3' |
| T6 forward primer | Primer | 5'-GATCGATCTCGCCCGCGAAATTAATACGACTCACTATA GGACAGCGCGTGTAG-3' |
| T6 reverse primer | Primer | 5'-CTAGCAGCGACGGAG-3' |
| T5 forward primer | Primer | 5'-GATCGATCTCGCCCGCGAAATTAATACGACTCACTATA GGATGAGCGACGCTG-3' |
| T5 reverse primer | Primer | 5'-GAGCAGTCGCCAGAC-3' |

| | | |
|---|---|---|
| T7 forward primer | Primer | 5'-GATCGATCTCGCCCGCGAAATTAATACGACTCACTATA GGCACCGTGGACACAG-3' |
| T7 reverse primer | Primer | 5'-CTCGACCTGGCAGTC-3' |
| T8 forward primer | Primer | 5'-GATCGATCTCGCCCGCGAAATTAATACGACTCACTATA GGCCTGCAAGTGCGAC-3' |
| T8 reverse primer | Primer | 5'-CACGTCGGTGGTCTC-3' |
| T4 forward sequencing primer 1 | Sequencing primer | 5'-AATGATACGGCGACCACCGAGATCTACACTC TTTCCCTACACGACGCTCTTCCGATCTNNN CTAGCT GGCACTGCAGAGTCG-3' |
| T4 forward sequencing primer 2 | Sequencing primer | 5'-AATGATACGGCGACCACCGAGATCTACACTCTTT CCCTACACGACGCTCTTCCGATCTNNN GACGAC GGCACTGCAGAGTCG-3' |
| T4 reverse sequencing primer | Sequencing primer | 5'-CAAGCAGAAGACGGCATACGAGATCGGTCTCGG CATTCCTGCTGAACCGCTCTTCCGATCT GCTCTGGCTCGAGTC-3' |
| T6 forward sequencing primer 1 | Sequencing primer | 5'-AATGATACGGCGACCACCGAGATCTACACTCTTT CCCTACACGACGCTCTTCCGATCTNNN CTCAGA GGACAGCGCGTGTAG-3' |
| T6 forward sequencing primer 2 | Sequencing primer | 5'-AATGATACGGCGACCACCGAGATCTACACTCTTT CCCTACACGACGCTCTTCCGATCTNNN TACAGC GGACAGCGCGTGTAG-3' |
| T6 reverse sequencing prime | Sequencing primer | 5'-CAAGCAGAAGACGGCATACGAGATCGGTCTCGGCA TTCCTGCTGAACCGCTCTTCCGATCT CTAGCAGCGACGGAG-3' |
| T5 forward sequencing primer 1 | Sequencing primer | 5'-AATGATACGGCGACCACCGAGATCTACACTCTTT CCCTACACGACGCTCTTCCGATCT NNN GGTAGC GGATGAGCGACGCTG-3' |
| T5 forward sequencing primer 2 | Sequencing primer | 5'-AATGATACGGCGACCACCGAGATCTACACTCTTT CCCTACACGACGCTCTTCCGATCT NNN ACTGAT GGATGAGCGACGCTG-3' |

| | | |
|---|---|---|
| T5 forward sequencing primer 3 | Sequencing primer | 5'-AATGATACGGCGACCACCGAGATCTACACTCTTT CCCTACACGACGCTCTTCCGATCT NNN ATGAGC GGATGAGCGACGCTG-3’ |
| T5 forward sequencing primer 4 | Sequencing primer | 5'-AATGATACGGCGACCACCGAGATCTACACTCTTT CCCTACACGACGCTCTTCCGATCT NNN ATTCCT GGATGAGCGACGCTG-3’ |
| T5 forward sequencing primer 5 | Sequencing primer | 5'-AATGATACGGCGACCACCGAGATCTACACTCTTT CCCTACACGACGCTCTTCCGATCT NNN CAAAAG GGATGAGCGACGCTG-3’ |
| T5 reverse sequencing primer | Sequencing primer | 5'-CAAGCAGAAGACGGCATACGAGATCGGTCTC GGCATTCCTGCTGAACCGCTCTTCCGATCT GAGCAGTCGCCAGAC-3’ |
| T7 forward sequencing primer 1 | Sequencing primer | 5'-AATGATACGGCGACCACCGAGATCTACACTCTTT CCCTACACGACGCTCTTCCGATCTNNN TATAAT GCACCGTGGACACAG-3’ |
| T7 forward sequencing primer 2 | Sequencing primer | 5'-AATGATACGGCGACCACCGAGATCTACACTCTTT CCCTACACGACGCTCTTCCGATCTNNN TCATTC GCACCGTGGACACAG-3’ |
| T7 reverse sequencing primer | Sequencing primer | 5'-CAAGCAGAAGACGGCATACGAGATCGGTCTCGG CATTCCTGCTGAACCGCTCTTCCGATCT CTCGACCTGGCAGTC-3’ |
| T8 forward sequencing primer 1 | Sequencing primer | 5'-AATGATACGGCGACCACCGAGATCTACACTCTTT CCCTACACGACGCTCTTCCGATCTNNN TCCCGA GCCTGCAAGTGCGAC-3’ |
| T8 forward sequencing primer 2 | Sequencing primer | 5'-AATGATACGGCGACCACCGAGATCTACACTCTTT CCCTACACGACGCTCTTCCGATCTNNN TCGAAG GCCTGCAAGTGCGAC-3’ |
| T8 reverse sequencing primer | Sequencing primer | 5'-CAAGCAGAAGACGGCATACGAGATCGGTCTCGG CATTCCTGCTGAACCGCTCTTCCGATCT CACGTCGGTGGTCTC-3' |
| | | |

| | | |
|---|---|---|
| T5R8/359 | T5 round 8 | 5’- GGATGAGCGACGCTG<br>GAAGGAAGAAAATGCAGAAAAAAAGAAAAAAATGTCTGG<br>GTCTGGCGACTGCTC-3’ |
| T5R8/359Δ6 | T5 without 3' primer insert | 5’- GGATGAGCGACGCTG<br>AAAAAAAAAAAAAAAAAAAAAAAAAAAAAAAAAGTCTGG<br>GTCTGGCGACTGCTC-3’ |
| 631 (T5R8/359 without 5' primer) | T5R8/359 without 5' primer | 5’- GAGCAGTCGCCAGAC<br>CCAGACATTTTTTTCTTTTTTTCTGCATTTTCTTCCTTC<br>TATAGTGAGTCGTATTAATTTC-3’ |
| 632 (T5R8/359 without 3' primer) | T5R8/359 without 3' primer | 5’- CCAGACATTTTTTTCTTTTTTTCTGCATTTTCTTCCTTC<br>CAGCGTCGCTCATCCTATAGTGAGTCGTATTAATTTC-3’ |
| T7R12/395 | T7 round 12 | 5’-GCACCGTGGACACAG<br>AAAAAAGAAAGGTAAGATTAATACGACATACCTTCCACT<br>GTGGACTGCCAGGTCGAGAG-3' |
| T7 R16/398 | T7 round 12 | 5’-GCACCGTGGACACAG<br>TGGATAAAGGAAGCATATGAAACTACAGCCCTCTATTTC<br>CCTGTGGACTGCCAGGTCGAG-3' |
| T7R16/399 | T7 round 16 | 5’ -GCACCGTGGACACAG<br>GAAGGGACCAGAAAGAGTGGCTAAGAAAGAGTCCCCTG<br>TGACTGCCAGGTCGAG-3' |
| T7R16/400 | T7 round 16 | 5’ -GCACCGTGGACACAG<br>GAAAAAGAAGAGAGCGCAAAGAAAGGAAATGTCTCCCT<br>GTGACTGCCAGGTCGAG-3' |
| T7R16/401 | T7 round 16 | 5’-GCACCGTGGACACAG<br>AAGATAAGGGAGAAAAATGGAACTGCGACCCACTATATC<br>TCTGTGGACTGCCAGGTCGAG-3' |
| T8R12/402 | T8 round 12 | 5’-GCCTGCAAGTGCGAC<br>CAAGGGA**GGAAGAAAATG**AAGAGGGAGACCTTCTACG<br>GGAGACCACCGACGTG-3’ |

| | | |
|---|---|---|
| T8R16/409 | T8 round 16 | 5'-GCCTGCAAGTGCGAC GAATGGGAAATAGCAAAAGAAAACAGAGACACCGACAT GTTAGAGACCACCGACGTG-3' |
| T8R16/410 | T8 round 16 | 5'-GCCTGCAAGTGCGAC TCACTGAATGGGAAGATGTGATGATGAGCCCTCCTACGT GAGACCACCGACGTG-3' |
| 620 (T8R16/409 5' deletion) | T8R16/409 without 5' primer | 5'-CACGTCGGTGGTCTC TAACATGTCGGTGTCTCTGTTTTCTTTTGCTATTTCCCAT TCTATAGTGAGTCGTATTAATTTC-3' |
| 621 (T8R16/409 3' deletion) | T8R16/409 without 3' primer | 5'-TAACATGTCGGTGTCTC TGTTTTCTTTTGCTATTTCCCATTCGTCGCACTTGCAGGC TATAGTGAGTCGTATTAATTTC-3' |
| 630 (T8R16/409 without 5' and 3') | T8R16/409$_{core}$ (T8R16/409 without 5' and 3' primer | 5'-TAACATGTCGGTGTCTC TGTTTTCTTTTGCTATTTCCCATTC TATAGTGAGTCGTATTAATTTC-3' |
| 622 (T8R16/409 without nt 30-52) | T8R16/409 without nt 30-52 (core deletion) | 5'-CACGTCGGTGGTCTC TAACCCGAAGGCTATTTCCCATTCGTCGCACTTGCAGGC TATAGTGAGTCGTATTAATTTC-3' |
| TxT7 | T7 RNA pol primer sequence | 5'-GATCGAGATCTCGATCCCGCGAAATTAATACGACTCAC TATA-3' |
| T4R20/372 | T4 R20 | 5'-GGCACTGCAGAGTCG TTATAGACTCACTATTATTTATCGCTCGTCTCCTCGTTGG ACTCGAGCCAGAGC-3' |
| T4R20/373 | T4 R20 | 5'-GGCACTGCAGAGTCG TAATTCCCTTTTTCTGCTCTACTTCTTTCTCTTTGGGTGG ACTCGAGCCAGAGC-3' |
| T4R20/375 | T4 R20 | 5'-GGCACTGCAGAGTCG TAATTCCCTTTTTCGCTCTATTTCCTTTTCTTTGGGTGGA CTCGAGCCAGAGC-3' |

| | | |
|---|---|---|
| T4R24/377 | T4 R24 | 5'-GGCACTGCAGAGTCG TCCTATGACTCATTTATTTTTATCGACTCGTTTCCCTGTG GACTCGAGCCAGAGC-3' |
| T4R24/378 | T4 R24 | 5'-GGCACTGCAGAGTCG TTATAGACTCACTATTATTTATCGCTCGTCTCCTCGTTGG ACTCGAGCCAGAGC-3' |
| T4R24/379 | T4 R24 | 5'-GGCACTGCAGAGTCG TATAGACTCACTATTATTTATCGCTCGTCTCCTCGTTGGA CTCGAGCCAGAGC-3' |
| T6R20/382 | T6 R20 | 5'-GGACAGCGCGTGTAG CCTTTTATTTTTCTCTCTCTGCTCAGGCTAGTACGCTCCT CCGTCGCTGCTAG-3' |
| T6R20/384 | T6 R20 | 5' -GGACAGCGCGTGTAG CCTTTTATTTTTCCCTCTCTGCTCAGGCTAGTGCGCTCCT CCGTCGCTGCTAG-3' |
| T6R24/387 | T6 R24 | 5'-GGACAGCGCGTGTAG ACCTTTTATTTTTCGGCTCTTCATAGGTCCTTCCCGCCTG CTCCGTCGCTGCTAG-3' |
| T6R24/388 | T6 R24 | 5'-GGACAGCGCGTGTAG AGTTTTTACTTTCGCTGCCTTTTCTCTACTTTCCGCTGCT CCTCCGTCGCTGCTAG-3' |
| T6R24/391 | T6 R24 | 5'-GGACAGCGCGTGTAG TGATGTTCATCGGAGCCACCCTTTTATCACACGCTGCTC CTCCGTCGCTGCTAG-3' |
| 105 (T8R16 / 409core) | Chemically synthesized RNA | 5'-Cy5-iSP18GCCUGCAAGUGCGACGAAUGGGAAAUAGC AAAAGAAAACAGAGACACCGACAUGUUAGAGACCACCG ACGUG-3' |
| T5R8/359 | Chemically synthesized RNA for ITC | 5'- GGAUGAGCGACGCUG GAAGGAAGAAAAUGCAGAAAAAAAGAAAAAAAUGUCUG G GUCUGGCGACUGCUC-3' |
| T8R16/409 | Chemically synthesized RNA for ITC | 5'-GCCUGCAAGUGCGAC GAAUGGGAAAUAGCAAAAGAAAACAGAGACACCGACAU GUUAGAGACCACCGACGUG-3' |

**Supplementary Table 2. Column binding and elution assay**

Analysis of PAGE gels from column binding assays (ATP agarose) of different RNA sequences (see Supplementary Table 1). Values (in %) represent the amount of RNA for each individual fraction on the gel, data were generated from 2-3 experimental repeats. * Fractions 3-5 are combined.

| | T5 | T5R8/359Δ6 (T5R8/359 without 3' primer insert) | T5R8/359 |
|---|---|---|---|
| unbound | 91.7 | 93.4 | 32.8 |
| wash | 5.0 | 2.1 | 7.5 |
| ATP 1 | 1.0 | 1.6 | 44.6 |
| ATP 2 | 0.9 | 1.1 | 7.6 |
| ATP 3 | 1.3 | 1.8 | 7.5* |

| | T7 | T7R12/ 395 | T7R16/ 401 | T7R16/ 400 | T7 R16/ 398 | T7R16/ 399 |
|---|---|---|---|---|---|---|
| unbound | 92.0 | 86.5 | 87.5 | 88.3 | 89.0 | 89.4 |
| wash | 4.8 | 4.5 | 2.1 | 2.4 | 2.9 | 5.1 |
| ATP 1 | 1.6 | 4.1 | 4.7 | 3.6 | 2.1 | 2.6 |
| ATP 2 | 1.3 | 4.4 | 3.9 | 3.7 | 3.6 | 1.5 |
| ATP 3 | 0.3 | 0.4 | 1.8 | 2.1 | 2.4 | 1.4 |

| | T8 | T8R12/ 402 | T8R16/ 410 | T8R16/ 409 | T8R16/ 409 |
|---|---|---|---|---|---|
| unbound | 94.9 | 84.5 | 91.9 | 35.1 | 32.7 |
| wash | 3.8 | 5.3 | 3.7 | 6.7 | 6.4 |
| ATP 1 | 0.0 | 7.9 | 1.9 | 27.7 | 20.9 |
| ATP 2 | 0.1 | 2.2 | 1.9 | 19.1 | 14.4 |
| ATP 3 | 1.1 | 0.1 | 0.6 | 11.4 | 11.8 |
| ATP 4 | | | | | 4.6 |
| ATP 5 | | | | | 5.7 |
| ATP 6 | | | | | 3.5 |

| | T4 | T4R20/ 372 | T4R20/ 373 | T4R20/ 375 | T4R24/ 377 | T4R24/ 378 | T4R24/ 379 |
|---|---|---|---|---|---|---|---|
| unbound | 92.0 | 87.5 | 90.2 | 95.6 | 84.3 | 93.8 | 89.5 |
| wash | 5.1 | 3.3 | 3.4 | 1.0 | 3.4 | 3.5 | 4.8 |
| ATP 3 | 0.8 | 5.6 | 1.9 | 2.1 | 6.9 | 1.2 | 3.5 |
| ATP 2 | 1.1 | 1.1 | 2.4 | 0.2 | 2.7 | 0.8 | 1.8 |
| ATP 3 | 0.9 | 2.5 | 2.1 | 1.2 | 2.6 | 0.7 | 0.4 |

| | T6 | T6R20/ 382 | T6R20/ 384 | T6R20/ 387 | T6R24/ 391 | T6R24/ 388 |
|---|---|---|---|---|---|---|
| unbound | 92.3 | 89.2 | 91.3 | 92.9 | 92.3 | 83.3 |
| wash | 3.6 | 4.6 | 3.0 | 3.4 | 5.3 | 2.8 |
| ATP 1 | 1.0 | 3.2 | 2.3 | 2.2 | 1.1 | 5.8 |
| ATP 2 | 0.7 | 1.8 | 2.0 | 0.9 | 0.5 | 6.4 |
| ATP 3 | 2.4 | 1.2 | 1.4 | 0.6 | 0.9 | 1.8 |

**Supplementary Table 3. Column binding and elution assay of GTP aptamer variants**

Analysis of PAGE gels from column binding assays (ATP agarose) of different RNA sequences (see Supplementary Table 1). Values (in %) represent the amount of RNA for each individual fraction on the gel, data were generated from 2-3 experimental repeats. * Fractions 3-5 are combined.

| | 620 (T8R16/409 without 5' primer) | 621 (T8R16/409 without 5' primer) | 630 (T8R16/ 409core) | 622 (T8R16/409 without core) | 105 (T8R16/409 core RNA) |
|---|---|---|---|---|---|
| unbound | 47.6 | 47.6 | 59.7 | 90.7 | 35.2 |
| wash | 8.4 | 12.8 | 6.9 | 4.6 | 2.3 |
| ATP 1 | 26.1 | 20.2 | 11.7 | 3.6 | 27.5 |
| ATP 2 | 11.3 | 11.5 | 14.0 | 0.3 | 20.6 |
| ATP 3 | 6.5 | 7.9 | 7.7 | 0.8 | 14.4* |

| | 105 (buffer elution) | 105 (ATP elution) | 105 (CTP elution) | 105 (GTP elution) | 105 (UTP elution) |
|---|---|---|---|---|---|
| unbound | 84.2 | 24.1 | 34.5 | 29.9 | 34.7 |
| wash | 5.3 | 7.5 | 6.4 | 6.3 | 9.9 |
| ATP 1 | 3.2 | 29.8 | 18.5 | 46.9 | 9.9 |
| ATP 2 | 2.3 | 20.6 | 16.8 | 9.6 | 21.3 |
| ATP 3 | 1.8 | 9.2 | 10.9 | 3.6 | 14.4 |
| ATP 4 | 1.7 | 5.8 | 7.7 | 2.3 | 3.6 |
| ATP 5 | 1.5 | 3.0 | 5.3 | 1.4 | 6.1 |

## References

[16] R. Lorenz, S. H. Bernhart, C. H. Z. Siederdissen, H. Tafer, C. Flamm, P. F. Stadler, I. L. Hofacker, *Algorithm. Mol. Biol.* **2011**, *6*.
[22] L. van der Maaten, G. Hinton, *Journal of Machine Learning Research* **2008**, *9*, 2579-2605.
